# Human induced pluripotent stem cell‐derived lung organoids in an ex vivo model of the congenital diaphragmatic hernia fetal lung

**DOI:** 10.1002/sctm.20-0199

**Published:** 2020-09-19

**Authors:** Shaun M. Kunisaki, Guihua Jiang, Juan C. Biancotti, Kenneth K. Y. Ho, Briana R. Dye, Allen P. Liu, Jason R. Spence

**Affiliations:** ^1^ Department of Surgery Johns Hopkins University Baltimore Maryland USA; ^2^ Institute for Cell Engineering, Johns Hopkins University Baltimore Maryland USA; ^3^ Department of Surgery University of Michigan Ann Arbor Michigan USA; ^4^ Department of Mechanical Engineering University of Michigan Ann Arbor Michigan USA; ^5^ Department of Cell and Developmental Biology University of Michigan Ann Arbor Michigan USA; ^6^ Department of Internal Medicine University of Michigan Ann Arbor Michigan USA

**Keywords:** congenital diaphragmatic hernia, fetal lung, induced pluripotent stem cells, lung organoids, mechanical compression

## Abstract

Three‐dimensional lung organoids (LOs) derived from pluripotent stem cells have the potential to enhance our understanding of disease mechanisms and to enable novel therapeutic approaches in neonates with pulmonary disorders. We established a reproducible ex vivo model of lung development using transgene‐free human induced pluripotent stem cells generated from fetuses and infants with Bochdalek congenital diaphragmatic hernia (CDH), a polygenic disorder associated with fetal lung compression and pulmonary hypoplasia at birth. Molecular and cellular comparisons of CDH LOs revealed impaired generation of NKX2.1^+^ progenitors, type II alveolar epithelial cells, and PDGFRα^+^ myofibroblasts. We then subjected these LOs to disease relevant mechanical cues through ex vivo compression and observed significant changes in genes associated with pulmonary progenitors, alveolar epithelial cells, and mesenchymal fibroblasts. Collectively, these data suggest both primary cell‐intrinsic and secondary mechanical causes of CDH lung hypoplasia and support the use of this stem cell‐based approach for disease modeling in CDH.

## Graphical abstract

We established a reproducible ex vivo model of lung development using transgene‐free human induced pluripotent stem cells generated from fetuses and infants with Bochdalek congenital diaphragmatic hernia (CDH). Both primary cell‐intrinsic and secondary causes of CDH lung hypoplasia were identified, and mechanical compression was associated with alterations in lung organoid epithelial and mesenchymal gene regulation.

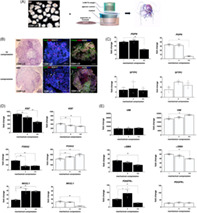

**Significance statement**

This study established a reproducible ex vivo model of lung development using transgene‐free human induced pluripotent stem cells generated from fetuses and infants with Bochdalek congenital diaphragmatic hernia (CDH). Both primary cell‐intrinsic and secondary causes of CDH lung hypoplasia were identified, and mechanical compression was associated with alterations in lung organoid epithelial and mesenchymal gene regulation.

## 1 INTRODUCTION

Bochdalek congenital diaphragmatic hernia (CDH), a condition associated with pulmonary hypoplasia at birth, is one of the most common, costly, and severe birth defects managed by neonatologists and pediatric surgeons worldwide.^1^, ^2^ The overall mortality rate in neonates born with CDH is 30%, and there is a wide spectrum of morbidity based largely on the degree of pulmonary disease.^3^ In those born with the severe disease phenotype, state‐of‐the‐art advances in critical care, including novel pharmacologic targets, nonconventional ventilator strategies, extracorporeal membrane oxygenation, and fetal tracheal occlusion surgery, have failed to make a substantial impact in terms of improving outcomes.^4^, ^5^, ^6^ Moreover, among survivors with CDH, there are often chronic, long‐term problems that persist for years, as validated by the spawning of multidisciplinary clinics dedicated to the care of these patients at major children's hospitals.^7^, ^8^ A better understanding of disease pathogenesis of the CDH lung is needed if further inroads are to be made in the management of this debilitating condition.

The underlying cause of CDH remains unknown.^9^ Although family cohorts with an autosomal dominant inheritance have been reported, the vast majority of cases are sporadic,^10^ and no single genetic mutation accounts for more than 1% to 2% of cases.^11^, ^12^ Through molecular cytogenetic analyses, studies have identified copy number variants and chromosomal regions through which over 50 candidate CDH‐causative genes have been proposed in humans.^13^, ^14^, ^15^ Functional analyses of these genes using animal models have been difficult due to early embryonic lethality, lack of conditional mutants, and low/variable recapitulation of the CDH phenotype.^15^, ^16^

Physical environmental factors have increasingly been shown to regulate lung organogenesis.^17^ In the case of CDH, there has been a long‐standing belief that the associated lung abnormalities are primarily caused by mechanical compression of the developing lung upon failed closure of one of the pleuroperitoneal canals at 8 weeks gestation.^18^ The abdominal contents, usually liver and intestines, subsequently herniate through the diaphragmatic defect in utero, causing mass effect during the pseudoglandular and canalicular stages of pulmonary development. Evidence in support of a mechanical mechanism has been shown experimentally through surgical creation of a diaphragmatic defect in fetal lambs, which universally induces lung hypoplasia at term.^19^ Clinical studies of children with CDH have also shown that the volume of abdominal herniation in the fetus significantly correlates with the severity of lung hypoplasia and clinical disease.^20^

The classic paradigm of mechanical compression‐induced lung hypoplasia has been challenged by a number of investigators based on several important observations.^21^, ^22^, ^23^, ^24^ First, despite the fact that the diaphragmatic defect is almost always limited to only one of the hemi‐diaphragms, the observed pulmonary hypoplasia and pulmonary hypertension also affects the contralateral lung, albeit to a lesser degree.^9^ Second, other space‐occupying lesions where there is extrinsic mass effect on early fetal lung development (eg, congenital lung malformations) are not typically associated with significant lung hypoplasia and pulmonary hypertension at birth.^24^ Third, teratogenic rodent models of CDH (eg, nitrofen, bisdiamine) have suggested that abnormal lung morphogenesis starts as early as E11, either in the absence of any diaphragmatic defect or before the diaphragm has completely formed at E16.^25^, ^26^ Taken together, these observations suggest that there may be primary cell‐based defects in CDH lung morphogenesis that are merely exacerbated by exposure to mechanical compression forces.^27^

Given these fundamental questions regarding the embryologic origins of CDH pulmonary hypoplasia, we sought to develop a human stem cell‐based strategy for modeling fetal lung development and for determining the future potential for cell‐based treatment strategies in CDH. Our approach involved the directed differentiation of human induced pluripotent stem cells (iPSCs) from CDH patients into three‐dimensional (3D) lung organoids (LOs) containing both pulmonary epithelial and mesenchymal cell types. Based on results from CDH LOs generated from clones derived from six affected patients, these LOs possessed airway‐like epithelial structures, alveolar cell types, and mesenchyme most closely corresponding to the pseudoglandular and early canalicular stages of lung development. In the absence of mechanical forces, comparisons of CDH LOs with those derived from normal (non‐CDH) controls revealed impaired expression of NKX2.1^+^ lung progenitors, type II alveolar epithelial cells, and platelet derived growth factor receptor‐alpha^+^ (PDGFRα) myofibroblasts.

To recapitulate the lung compression that occurs in utero, we then applied mechanical forces to CDH organoids during the pseudoglandular stage of differentiation. We observed significant downregulation of *NKX2.1* as well as *SOX2* and *SOX9*, genes associated with proximal and distal lung progenitor differentiation, respectively. Mechanical compression also correlated with significant upregulation of pro‐fibrotic mesenchymal fibroblast genes. Collectively, these data support the notion of both primary cell‐intrinsic and secondary mechanical causes in the maldevelopment of the CDH lung. Further study of this research platform will be instrumental for elucidating the disease‐relevant mechanical cues and critical epithelial‐mesenchymal interactions that contribute to early lung development in CDH.

## 2 MATERIALS AND METHODS

### 2.1 Human tissue

De‐identified human amniotic fluid and neonatal foreskin samples were obtained by consent under approved institutional review board protocols at the Johns Hopkins University (IRB #202082) and the University of Michigan (IRB #38565). Amniotic fluid samples (8‐10 mL) were obtained by amniocentesis from confirmed cases of CDH by prenatal ultrasound (n = 2) as well as from normal fetuses without CDH (n = 2) at 18 to 24 weeks gestation (Table S1).^28^ Foreskin specimens were also obtained from male CDH (n = 4) and normal (n = 2) infants following elective circumcision. Isolation of somatic cells was performed as previously described in our laboratory.^28^, ^29^

As controls, blocks of human normal and CDH lung tissue (n = 4) from fetuses/neonates were obtained under the same institutional review board approval at the University of Michigan.

### 2.2 iPSC derivation

All work was carried out under oversight committee approval (HPSCRO #1044). iPSCs were generated using nonintegrating, cytoplasmic Sendai Virus (CytoTune‐iPS Reprogramming Kit, Invitrogen, Waltham, Massachusetts) as previously described.^28^, ^29^ Embryoid bodies (EBs) were formed in suspension culture and analyzed using standard methods.^30^ Karyotyping was performed on iPSCs by Cell Line Genetics (Madison, Wisconsin). Individual staining and measurements were performed in triplicate.

### 2.3 LO generation

Differentiation of iPSCs into definitive endoderm and subsequently into ventral‐anterior foregut spheroids was carried out using a protocol as detailed elsewhere.^31^, ^32^ Briefly, iPSCs were treated with 100 ng/mL Activin A (R&D Systems, Minneapolis, Minnesota) and then incubated in foregut media: DMEM/F12 (Invitrogen) containing N2 and B27 (Thermo Fisher, Waltham, Massachusetts), 10 mM HEPES (Thermo Fisher), 200 ng/mL Noggin (R&D), 10 μM SB431542 (Stemgent, Cambridge, Massachusetts), 2 μM Chiron (Stemgent), 500 ng/mL FGF4 (R&D), and 1 μM SAG (Enzo Life Science, Farmingdale, New York) for 8 days. Cells (25 organoids/well) were resuspended in 50 μL Matrigel (BD, Franklin Lakes, New Jersey), and LOs were maintained in a 24‐well plate with 500 ng/mL FGF10 (R&D) until the study endpoint of 20, 40, or 60 days.

### 2.4 Compression testing

The compression device was based on work described elsewhere^33^ with some modifications (Figure 6A) and has been previously reported.^34^ Briefly, day 40 CDH LOs (n = 10 per drop) were encapsulated into 50 μL of Matrigel and placed onto 0.4 μm pore size polyster (PET) transwell membranes (Fisher Scientific, 07‐200‐170, Waltham, Massachusetts). CDH and normal LOs were overlaid with a 1% agarose gel cushion (Invitrogen, Ultra‐Pure Agarose, 16 500‐100) made from sterilized 24‐mm polydimethylsiloxane (PDMS) molds. A weighted object corresponding to a predetermined compression force (range, 0‐400 Pa) was applied for 48 hours prior to further analyses. Forces were chosen based on in vitro and in vivo work described elsewhere.^17^, ^35^

### 2.5 Histology and immunofluorescence

All samples were fixed in paraformaldehyde. Confirmation of alkaline phosphatase (AP) expression within iPSCs was performed using the AP substrate kit (Millipore, Burlington, Massachusetts) according to the manufacturer's instructions. Organoids and tissues were embedded in paraffin and sectioned at 5 μm prior to staining. In preparation for immunofluorescence, sections underwent antigen retrieval with pH 9.0 Tris/EDTA Buffer. Human CDH lung tissue blocks (n = 6) were obtained for comparative analysis from the University of Michigan Department of Pathology (courtesy of Dr Raja Rabah). Staining was carried out as previously described.^31^ The antibodies and dilutions used are shown in Table S4. Slides were imaged using a Nikon A1 confocal microscope (Melville, New York) or an Olympus IX71 epifluorescent microscope (Center Valley, Pennsylvania). Cell quantification based on confocal images was performed in a blinded fashion from at least five sections per LO using ImageJ with a cell counter plugin.^31^ The total number of cells was determined by DAPI^+^ nuclei with the same section.

### 2.6 RNA extraction and qPCR

RNA was extracted using the MagMAX‐96 Total RNA Isolation Kit (Life Technologies, Carlsbad, California) and MAG Max Express (Applied Biosystems, Grand Island, New York). RNA concentration was measured with NanoDrop 2000 (Thermo Fisher). Reverse transcription was conducted using the SuperScript VILO kit (Invitrogen) and quantitative real‐time polymerase chain reaction (RT‐PCR) was carried out using Fast SYBR Green Master Mix (Applied Biosystems) on an AB‐QuantStudio3 real time PCR machine (Thermo Fisher). The primer sequences can be found in Table S5. *GAPDH* was used as a reference gene to normalize target gene expression.

### 2.7 Microarray analysis

Microarray analyses, focused exclusively on a limited set of genes associated with lung development and the extracellular matrix (ECM), were performed on amniocytes, dermal fibroblasts, iPSCs, and organoids (d20‐d60) using Affy Plus (Affymetrix, Santa Clara, California). Lists of genes analyzed are shown in Tables S2 and S3. Sample processing, probing to the Human Gene 2.1 ST array platform (Affymetrix), and data analysis were performed by the University of Michigan DNA Sequencing Core Microarray Facility. Normalized expressions by robust multiple‐array averages were plotted using the heatmap.2 function from the gplots package in R (www.r-project.org) using default parameters. The Euclidean distance dissimilarity matrix and complete linkage method were used to generate heatmap images. After exclusion of genes that were not present, values were normalized and compared to results from human fetal lung.^36^

### 2.8 Statistical analysis

Quantitative analyses were presented as the mean ± SEM. Statistical comparisons were by the Mann‐Whitney test or analysis of variance with Dunnett or Bonferroni correction for multiple comparisons, as appropriate, using GraphPad Prism software (version 8, GraphPad, La Jolla, California). Results were considered to be statistically significant if *P* ≤ .05. For immunostaining and quantitative polymerase chain reaction (qPCR) of LOs, data were representative of at least three iPSC lines unless stated otherwise. Individual stainings and measurements were performed in triplicate. Additional details of statistical tests can be found in the figure legends.

## 3 RESULTS

### 3.1 Generation of pluripotent stem cells from human CDH patients

We isolated human somatic cells from either the amniotic fluid (n = 2) or foreskin (n = 4) of children with CDH (Table S1). To epigenetically reprogram these cells into iPSCs, we used nonintegrating Sendai virus (SeV). We confirmed that morphologically distinct CDH‐iPSCs (Figure S1A, middle) expressed high levels of AP comparable to those from ESCs and iPSCs derived from the same somatic tissue source in normal, non‐CDH patients (Figure S1B). Between 4 and 6 weeks after exposure to SeV reprogramming factors, individual clones were mechanically picked and successfully transferred onto Matrigel‐coated dishes for further expansion, subculture, and additional characterization. Immunofluorescence staining revealed uniformly high expression of NANOG, OCT4, SOX2, and SSEA3 within colonies (Figure S1B). These data were supported by significant upregulation of pluripotency‐specific genes, including *NANOG*, *OCT4*, and *SOX2*, in iPSC clones derived from CDH patients compared to those observed in respective parental somatic cells (*P* ≤ .01, Figure S2C).

Cytogenetic analyses verified normal karyotypes, suggesting that CDH‐iPSCs remained free from major nonclonal aberrations after continuous in vitro expansion for up to 20 passages (Figure S1C). To confirm that iPSC‐CDH cells were capable of tri‐lineage differentiation in vitro, CDH‐iPSCs exhibited robust EB formation as shown by the presence of derivatives from all three germ layers on immunohistochemistry (Figure S1D).

### 3.2 Human CDH induced pluripotent stem cells adopt fetal lung‐like characteristics

**FIGURE 1:**
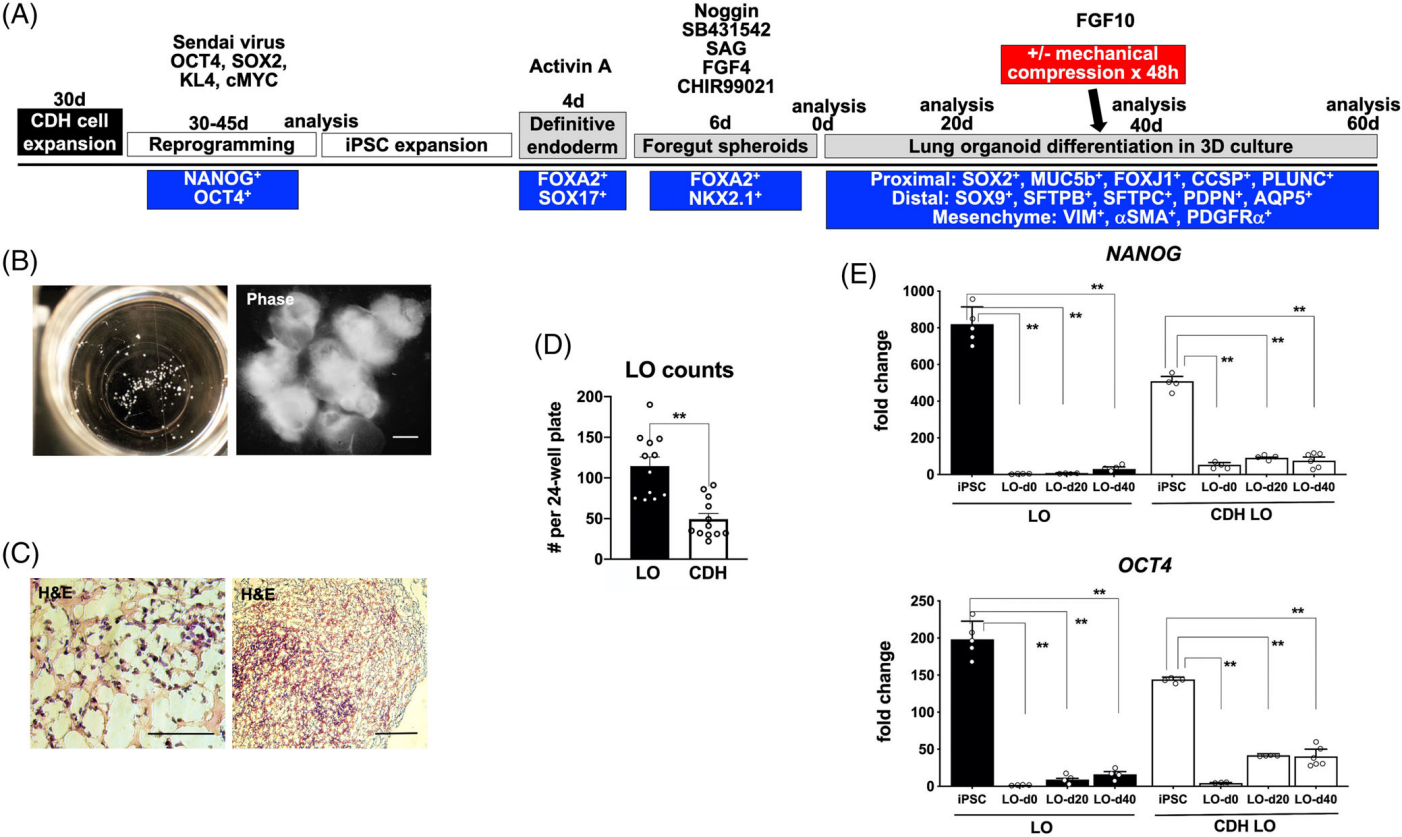
Human congenital diaphragmatic hernia (CDH) induced pluripotent stem cells (iPSCs) adopted characteristics of 3D lung‐like cells, termed lung organoids (LOs). A, Step‐wise schematic protocol for the generation of CDH foregut spheroids and LOs (n = 10 separate experiments) from iPSCs.^31^ All cells were analyzed at serial time points during organoid differentiation using phenotypic markers (shown in the blue boxes). Selected CDH LOs were also exposed to ex vivo mechanical compression (red box) at day 35 of differentiation to simulate the compression that occurs in vivo from herniated abdominal contents. B, Representative gross appearance of early CDH organoids (*left*) in a 24‐well plate (day 4 induction), each measuring 200 to 500 μm in diameter. Under higher magnification (*right*), phase contrast photomicrographs reveal the typical morphology of organoids derived from CDH iPSCs, magnification, ×5. Scale bar represents 50 μm. C, Representative histology of CDH LOs at day 20 (*left*, magnification, ×60) and day 60 (*right*, magnification, ×10) demonstrates the sac‐like architecture that resembles pulmonary alveolar‐like tissue (H&E staining). Scale bar represents 50 μm. D, Vertical bar graph with dot plots of day 28 organoid counts from typical 24‐well plates in normal and CDH LOs, ** denotes *P* ≤ .01 (Mann‐Whitney). E, Representative vertical bar graphs with dot plots of serial quantitative gene expression show significant downregulation of the pluripotency‐specific genes, *NANOG* and *OCT4*, in representative normal and CDH iPSCs clones (passage 22‐26) after foregut spheroid generation (LO‐d0) and during early 3D LO differentiation (LO‐d20 and LO‐d40). Data were normalized relative to housekeeping gene (*GAPDH*) and presented as the mean ± SEM, ** denotes *P* ≤ .01 compared to parental iPSCs (*left bars*, ANOVA), three independent biological replicates (three separate experiments using three different cell lines). Experiments performed without mechanical compression. ANOVA, analysis of variance

We then exposed human iPSC‐CDH clones to an established lung induction protocol (Figure 1A) based on manipulating fibroblast growth factor and hedgehog signaling as previously published.^31^ Ventral‐anterior foregut spheroids were generated from definitive endoderm in growth factor media containing FGF4, SAG, and CHIR99021, and subsequently cultured in Matrigel droplets. Spheroids grew robustly by day 4 (Figure 1B), demonstrating typical patterned morphology.^31^ Between 20 and 40 days of induction, ~30‐35 organoids per well could be generated, each with multiseptated, sac‐like architecture within the interior (Figure 1C). Organoid experiments were carried out for 20 (n = 6), 40 (n = 6), 60 (n = 3), or 100 (n = 1) days, and comparable results were obtained in each experiment using reprogrammed iPSCs clones from all six CDH patients (Table S1). The number of day 28 LOs was significantly decreased in CDH LOs compared to normal LOs (Figure 1D; 49.3 ± 7.0 vs 114.6 ± 11.2, respectively; *P* ≤ .01), consistent with possible lung hypoplasia in CDH. To confirm directed differentiation of human iPSC‐CDH clones into early LOs, we performed serial quantitative gene expression analyses at day 40 to determine degree of lung development. As expected, there were significant declines in *NANOG* and *OCT4* expression in CDH LOs and normal LOs (Figure 1E, *P* ≤ .01).

**FIGURE 2:**
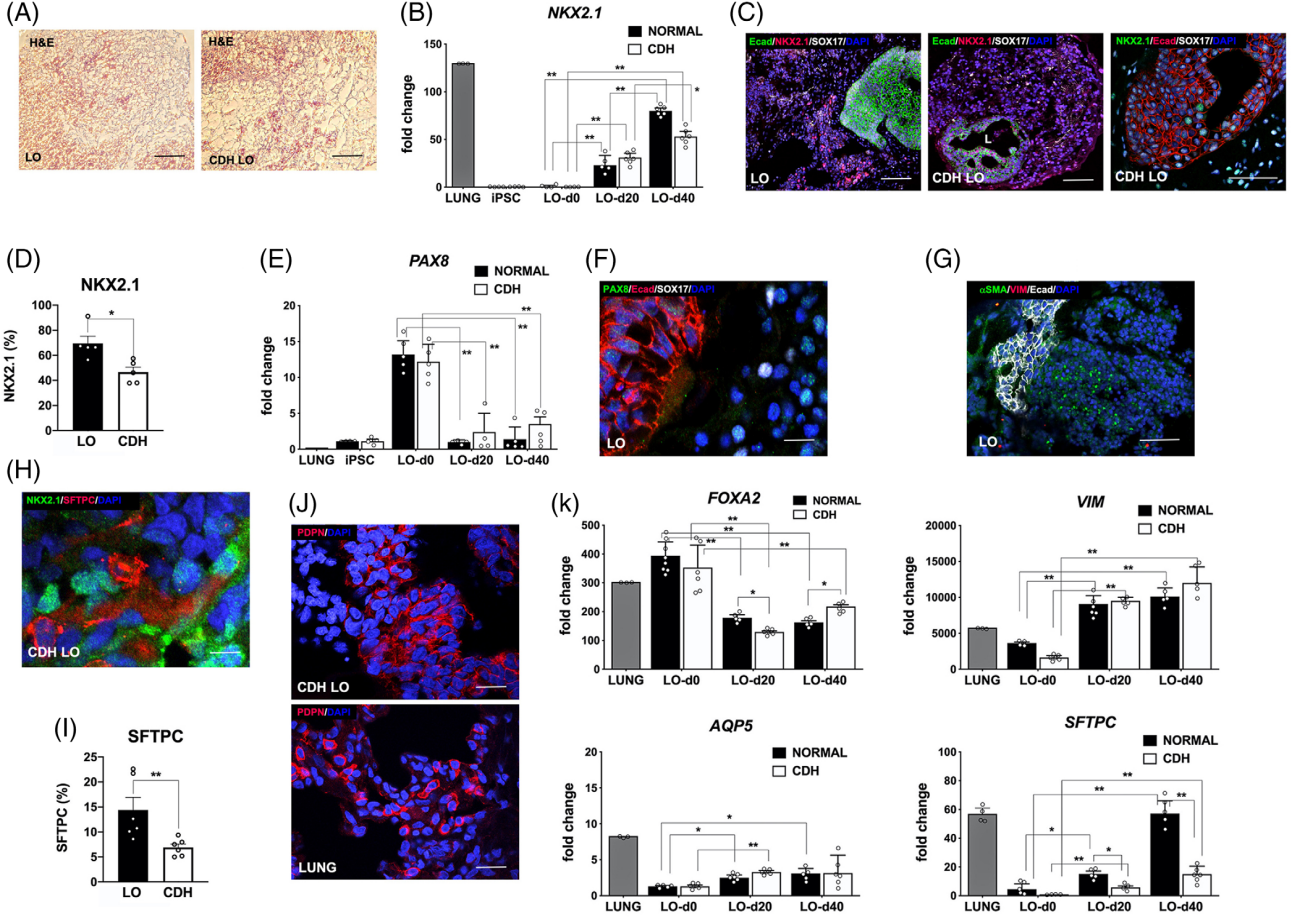
In the absence of mechanical compression, human lung organoids (LO) derived from induced pluripotent stem cells (iPSCs) of congenital diaphragmatic hernia (CDH) children revealed baseline impairments in lung differentiation compared to iPSCs of children with normal lungs. A, Representative histology of LOs at day 40 (magnification, ×20) in normal (*left*) and CDH (*right*) iPSCs by H&E staining. Scale bar represents 50 μm. B, Vertical bar graph with dot plots of the lung/anterior foregut progenitor gene, *NKX2.1*, shows significant progressive gene upregulation at day 20 and day 40. By day 40, there is a significant increase in LOs derived from children with normal lungs. * and ** denote *P* ≤ .05 and *P* ≤ .01 compared to normal control LOs (ANOVA), n = 4 to 6 per group. C, Representative confocal microscopy sections (magnification, ×10) of human LOs (day 40) derived from normal and CDH iPSCs reveal NKX2.1^+^ cells (TxRed secondary, red or FITC secondary, green) both within and adjacent to clusters of E‐cadherin (Ecad)‐positive staining. “L” indicates luminal structure. Scale bars represent 150 μm. D, Vertical bar graph with dot plots of NKX2.1 protein expression, expressed as a percentage of DAPI^+^ cells, shows significant downregulation in CDH LOs compared to normal LOs (69.5% ± 5.8% vs 46.6% ± 4.0%, respectively). * denotes *P* ≤ .05 (Mann‐Whitney). E, Vertical bar graph with dot plots of the anterior foregut progenitor gene, *PAX8*, shows minimal gene expression at day 20 and day 40, consistent with specific differentiation toward the pulmonary cell lineage. ** denotes *P* ≤ .01 (ANOVA), n = 4 to 6 per group. F, Representative confocal microscopy section (magnification, ×40) of human LOs (day 40) showing negligible PAX8^+^ expression (FITC, green). Scale bar represents 50 μm. G, Representative confocal microscopy section (magnification, ×10) indicate both epithelial and mesenchymal components within human LOs (day 40) as suggested by Ecad (Cy5 secondary, white) and αSMA (FITC, green) expression, respectively. Scale bar represents 200 μm. H, Representative confocal microscopy section (magnification, ×60) of CDH LOs (day 40) reveal both NKX2.1^+^ and type II alveolar epithelial marker, surfactant protein‐C^+^ (SFTPC) expression (FITC, green). Scale bar represents 10 μm. I, Vertical bar graph with dot plots of SFTPC protein expression shows significant downregulation in CDH LOs compared to normal LOs on day 20. ** denotes *P* ≤ .01 (Mann‐Whitney). J, Representative confocal microscopy sections (magnification, ×20) of CDH LOs (day 40, *top*) exhibit peripheral enhancement of cells with type I alveolar epithelial cell marker, podoplanin (PDPN^,^ TxRed, red) in similar pattern to human native lung tissue (*bottom*). Scale bars represent 25 μm. K, Vertical bar graphs with dot plots show expression of the endodermal gene, *FOXA2* (*upper left*), the mesenchymal gene, vimentin (*VIM*, *upper right*), and key lung epithelial genes, *AQP5* and *SFTPC* (*lower panels*).^37^ LO induction was associated with significant upregulation in both mesenchymal and lung epithelial genes. Significant impairment in SFTPC expression was noted among CDH LOs. Data were normalized relative to housekeeping gene (*GAPDH*) and presented as the mean ± SEM, * and ** denote *P* ≤ .05 and *P* ≤ .01 (ANOVA or Mann‐Whitney, as appropriate). Representative from three separate experiments using three different cell lines. Experiments performed without mechanical compression, and normal human fetal lungs provided as positive controls. ANOVA, analysis of variance; FITC, fluorescein isothiocyanate

We then conducted comparative analyses of normal and CDH LOs during the early lung differentiation phase by histology (Figure 2A), gene expression, and immunofluorescence. The expression of the lung/anterior foregut progenitor gene, *NKX2.1 (TTF‐1)*, was significantly upregulated in LOs at days 20 and 40, consistent with successful differentiation into the pulmonary lineage (*P* ≤ .01; Figure 2B). Moreover, NKX2.1 protein expression was documented on confocal microscopy (Figure 2C) and was significantly increased in normal LOs compared with CDH LOs in quantitative analysis (Figure 2D, *P* ≤ .05). Low or negligible expression of *PAX8*, a thyroid lineage marker, in association with the LO induction protocol was confirmed (Figure 2E,F, *P* ≤ .01). Demonstration of both epithelial and mesenchymal cell populations within normal and CDH LOs was shown by immunofluorescent staining for E‐cadherin (Ecad) and α‐smooth muscle actin/vimentin, respectively (Figure 2G).

The presence of type II alveolar cells, as suggested by surfactant protein‐C (SFTPC) protein expression, was evident adjacent to NKX2.1^+^ cells by day 20 (Figure 2H,I, *P* ≤ .01). The presence of type I epithelial cells was supported by podoplanin (PDPN) staining in an alveolar‐like distribution along the periphery of cells in both normal and CDH LOs (Figure 2J). Furthermore, LOs showed significant downregulation of the endodermal gene, *FOXA2*, as well as a significant upregulation of the lung epithelial genes, *AQP5 and SFTPC* (*P* ≤ .05 and *P* ≤ .01, respectively; Figure 2K). The mesenchymal gene, vimentin (*VIM*), was also significantly upregulated during LO induction (*P* ≤ .01). Taken together, these results were consistent with the successful differentiation of pluripotent stem cells from CDH patients into multicellular LOs containing both pulmonary epithelial and mesenchymal derivatives.

**FIGURE 3:**
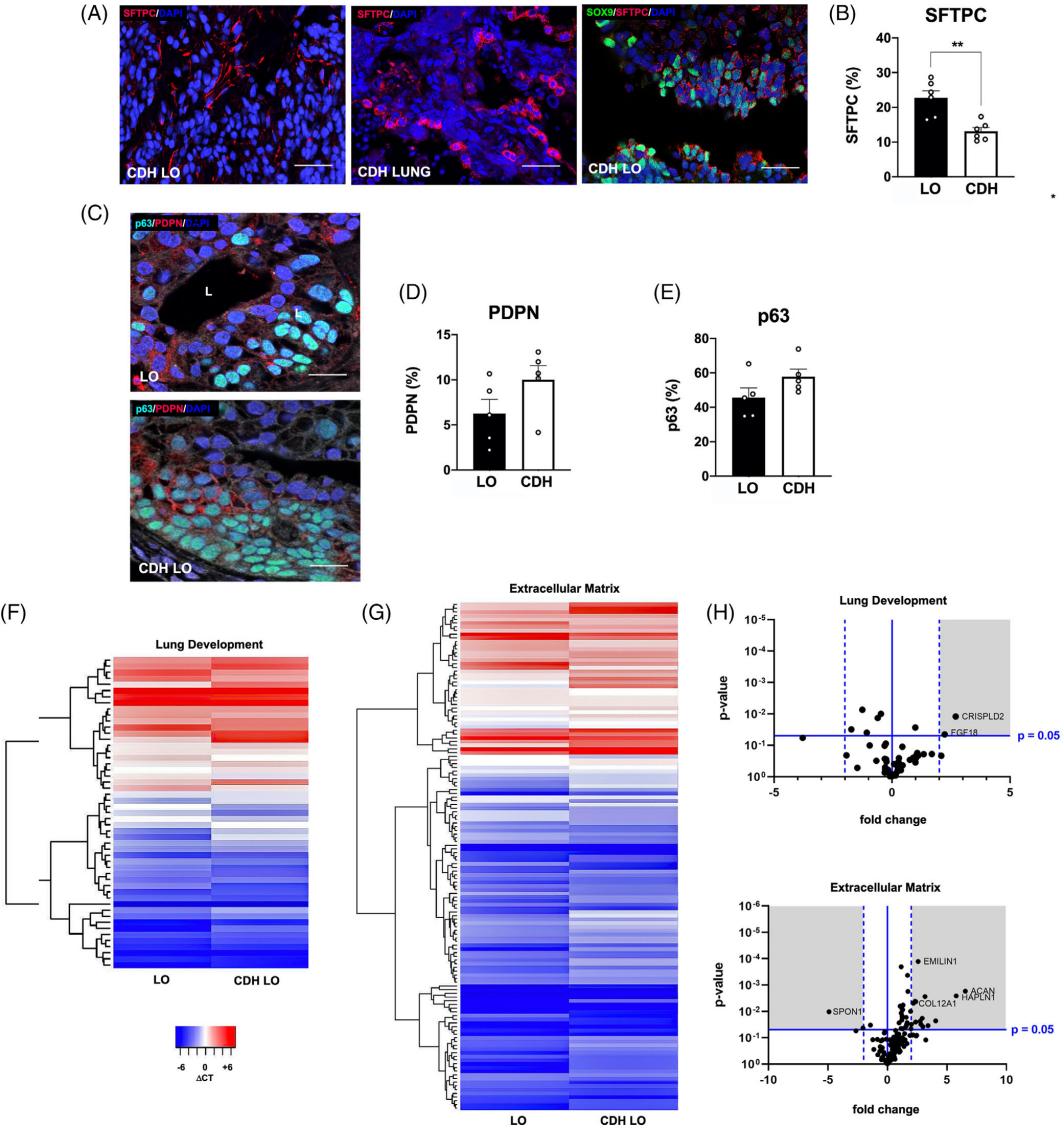
In the absence of mechanical compression, human lung organoids (LO) derived from induced pluripotent stem cells (iPSCs) of congenital diaphragmatic hernia (CDH) children showed differences in lung epithelial and mesenchymal differentiation. A, Representative confocal microscopy sections (magnification, ×40) of CDH LOs (day 40, *left*) indicate positive peripheral cell staining with surfactant protein‐C (SFTPC; TxRed secondary, red) in similar pattern to that of human native CDH lung tissue (*middle*). Further evidence of SFTPC lung epithelia is suggested by colocalization with distal epithelial marker, SOX9 (FITC secondary, green; *right*). Scale bars represent 50 μm. B, Vertical bar graph with dot plots of SFTPC protein expression on day 40 reveals significant downregulation in CDH LOs compared to normal LOs. ** denotes *P* ≤ .01 (Mann‐Whitney). C, Representative confocal microscopy sections (magnification, ×40) of CDH LOs (day 60, *top*) suggest positive peripheral cell staining with basal epithelial marker, p63 (FITC secondary, green) and PDPN (TxRed, red) in similar pattern to that of CDH LOs (*bottom*). Scale bars represent 25 μm. D,E, Vertical bar graphs with dot plots on day 40 reveal comparable levels of PDPN and p63 protein expression, respectively, in CDH LOs compared to levels in normal LOs. F, Microarray analysis produced a representative heatmap image documenting alterations in 54 lung development‐associated genes (Table S2), grouped based on hierarchical clustering, in day 40 LOs derived from an iPSC clone in a child with normal lungs (LO) compared with day 40 LOs derived from an iPSC clone in a child with CDH (CDH LO). Higher and lower levels of transcripts are shown in red and blue, respectively. Three independent biological replicates (three separate experiments using three different cell lines) without mechanical compression. G, Representative heatmap image shows changes in 99 ECM genes (Table S3), grouped based on hierarchical clustering, in day 40 LOs compared with day 40 CDH LOs. Higher and lower levels of transcripts are shown in red and blue, respectively. Three independent biological replicates (three separate experiments using three different cell lines) without mechanical compression, performed at both day 40 and day 60 time points. H, Volcano plot of differentially expressed 54 lung development‐associated genes in day 40 LOs derived from an iPSC clone in a child with CDH. Values are expressed as fold changes in CDH LOs compared with LOs derived from an iPSC clone in a child with normal lungs (shaded area denotes greater than twofold change, *P* ≤ .05). I, Volcano plot of differentially expressed 99 extracellular matrix‐associated genes in day 40 LOs derived from an iPSC clone in a child with CDH. Values are expressed as fold changes in CDH LOs compared with LOs derived from an iPSC clone in a child with normal lungs (shaded area denotes greater than twofold change, *P* ≤ .05). GEO link: https://www.ncbi.nlm.nih.gov/geo/query/acc.cgi?acc=GSE149780. ECM, extracellular matrix

To further assess proximal and distal airway progenitor cell differentiation within LOs, we then systematically analyzed LOs at day 40. There was intermittent but robust SFTPC staining throughout the periphery of cells (Figure 3A). Colocalization of SFTPC^+^ and SOX9^+^ cells was evident, supporting the differentiation of distal pulmonary epithelial cell types. Quantitative protein counts at day 40 confirmed significant decreases in SFTPC expression in CDH LOs compared to counts in normal LOs (Figure 3B, *P* ≤ .01). Double staining of LO sections showed the presence of the basal bronchial marker, p63, adjacent to cells with PDPN peripheral enhancement (Figure 3C). Previous work using this same induction protocol has shown that p63^+^ cells possess an NKX2.1^+^ identity.^31^ There were no significant differences in p63 and PDPN expression between normal and CDH LOs (Figure 3D,E).

### 3.3 Targeted microarray analyses reveal upregulation in ECM gene expression within human CDH LOs

To assess for similarities and differences in gene expression between CDH‐LOs and control LOs during organoid differentiation, we performed a microarray analysis using panels targeting genes associated with lung development and ECM activity (Tables S2 and S3, respectively). After normalization of gene expression relative to parental iPSCs, heatmaps of upregulated and downregulated genes in day 20, 40, and 60 CDH LOs were generated and compared to age‐matched normal LOs (Figure 3F,G). Volcano plots of differentially expressed lung development‐associated genes in day 40 CDH LOs revealed significant dysregulation in only two (1.9%) genes, fibroblast growth factor‐18 (*FGF18*) and cysteine rich secretory protein LCCL domain containing‐2 (*CRISPLD2*), relative to day 40 normal LOs (Figure 3H, greater than twofold change, *P* ≤ .05). No significant dysregulation was seen in day 20 or day 60 CDH LOs. Moreover, there were no differentially expressed genes associated with lung development, including markers of retinoic acid signaling (*STRA6*, *RBP4*), among CDH LOs evaluated (volcano plots not shown). Sonic hedgehog (*SHH*) was downregulated in day 40 CDH LOs, but this did not meet statistical significance (*P* = .058). In contrast, there were 15 (10.9%) ECM‐associated genes that were significantly upregulated in day 40 CDH LOs (Figure 3I), including hyaluronan and proteoglycan link protein‐1 (*HAPLN1*), aggrecan (*ACAN*), and elastin microfibril interface‐1 (*EMILIN1*, greater than twofold change, *P* ≤ .05).

### 3.4 Human CDH LOs show impairments in pulmonary epithelial and mesenchymal differentiation

**FIGURE 4:**
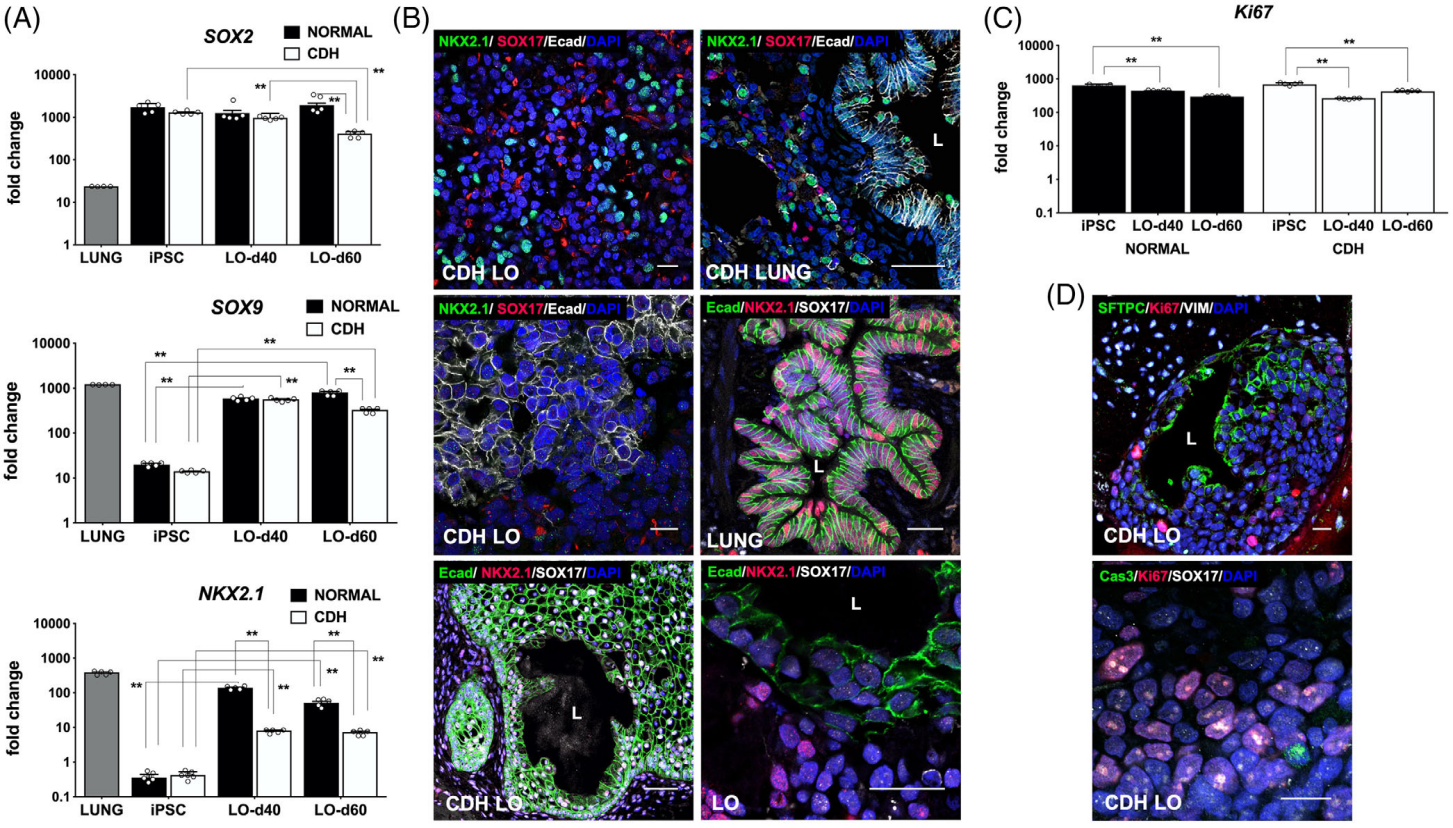
In the absence of mechanical compression, human lung organoids (LO) derived from induced pluripotent stem cells (iPSCs) of congenital diaphragmatic hernia (CDH) children showed differences in lung progenitor, proximal, and distal cell differentiation. A, Vertical bar graphs with dot plots of quantitative *SOX2* gene expression show continued upregulation in normal and CDH iPSCs clones for up to 60 days after LO induction (*upper panel*). There was also significant downregulation of the distal lung progenitor marker, *SOX9* (*middle*), and *NKX2.1* (*lower*) in CDH LOs compared to parental iPSC controls. Data were normalized relative to housekeeping gene (*GAPDH*) and presented as the mean ± SEM, ** denotes *P* ≤ .01 compared to parental iPSCs (Mann‐Whitney), three independent biological replicates (three separate experiments using three different cell lines) without mechanical compression, and normal human fetal lungs were a positive control. B, Representative confocal microscopy sections (magnification, ×40) of human CDH LOs (day 40, *upper left*) exhibit lung progenitor cell immunostaining patterns with some similarity to human CDH lung (*upper right*), including NKX2.1^+^ lung progenitors (FITC secondary, green) and endodermal SOX17^+^ cells (TxRed secondary, red). Scale bars represent 10 μm. A robust E‐cadherin^+^ (Ecad) staining pattern (Cy5 secondary, white) was apparent in CDH LOs at day 40 (*middle left*, magnification, ×40) and day 60 (*lower left*; FITC secondary green; magnification, ×20) analogous to Ecad staining within tissue from infant CDH lungs (*upper right*; Cy5 secondary, white; magnification, ×40), tissue from normal infant lungs (*middle right*; FITC secondary, green; magnification, ×40), and day 60 LOs derived from children with normal lungs (*lower right*, magnification, ×60). Scale bars represent 25 μm. “L” indicates the lumen. C, Representative vertical bar graph of quantitative *Ki67* gene expression shows continued expression of this cellular proliferation marker in both normal and CDH iPSCs clones for up to 60 days after LO induction (*upper panel*). Data were normalized relative to housekeeping gene (*GAPDH*), presented as the mean ± SEM, * denotes *P* ≤ .01 compared to parental iPSC controls (Student's *t* test), and three independent biological replicates without mechanical compression (three separate experiments using three different cell lines). D, Representative confocal sections at day 40 show microscopic CDH LO lumen lined with SFTPC^+^ cells (FITC secondary, green) and Ki67^+^ cells (TxRed secondary, red) along its periphery and evidence of a vimentin (VIM)^+^ stroma (Cy5 secondary, white; *top*, magnification, ×20). High power image of CDH LO reveal robust Ki67^+^ cells (TxRed secondary, red) and endodermal SOX17^+^ cells (Cy5 secondary, white) with scant activated caspase 3 (Cas3)^+^ cells (FITC secondary, green; *bottom*, magnification, ×60). Scale bars represent 10 μm. “L” indicates the lumen

We also performed a comparative analysis of proximal and distal lung gene expression within normal and CDH LOs up to day 60 of lung differentiation. High gene expression of the proximal lung progenitor marker, *SOX2*, was maintained in iPSC‐LO clones from both CDH and normal patients for up to 60 days after induction but was significantly diminished in day 60 CDH LOs (Figure 4A, top; *P* ≤ .01). There was significant upregulation of the distal lung progenitor marker, *SOX9*, among LOs compared to that observed in baseline parental iPSCs (*P* ≤ .01). However, gene expression of *SOX9* was significantly lower at day 60 in CDH LOs compared to LOs derived from children with normal lungs (Figure 4A, middle; *P* ≤ .01). *NKX2.1* gene expression remained significantly impaired in day 60 CDH LOs (Figure 4A, bottom; *P* ≤ .01). Taken together, these data suggest possible impairment in the differentiation of human CDH‐LOs into NKX2.1^+^, SOX2^+^, and SOX9^+^ progenitors under identical conditions, even in the absence of mechanical compression.

We then directly compared how markers of pulmonary differentiation within human CDH‐LOs might differ from those in human perinatal lung tissue. Confocal microscopy images of CDH LOs exhibited similar lung progenitor cell immunostaining patterns comparable to those observed in infant CDH lung, including NKX2.1^+^ lung progenitors and endodermal SOX17^+^ cells (Figure 4B). In other areas adjacent to hollow lumen‐like structures, there was a robust staining for Ecad and a relative paucity of NKX2.1^+^ cells compared to that observed in native lung tissue and normal LO controls.

We also evaluated differences in proliferating cells within CDH LOs compared to normal LO controls. Expression of *Ki67*, a cellular proliferation gene, was maintained in all iPSCs throughout the LO induction phase (Figure 4C), but significant reductions were observed at later time points (*P* ≤ .01). There were no significant differences in *Ki67* gene expression between LOs from normal iPSCs and those derived from iPSCs‐CDH. Confocal sections demonstrated microscopic luminal structures lined with SFTPC^+^ cells and Ki67^+^ cells along the basal region (Figure 4D, top). There was scant evidence of the cellular apoptosis protein, activated caspase‐3 (Cas3), in normal and CDH LOs by immunofluorescence staining (Figure 4D, bottom).

**FIGURE 5:**
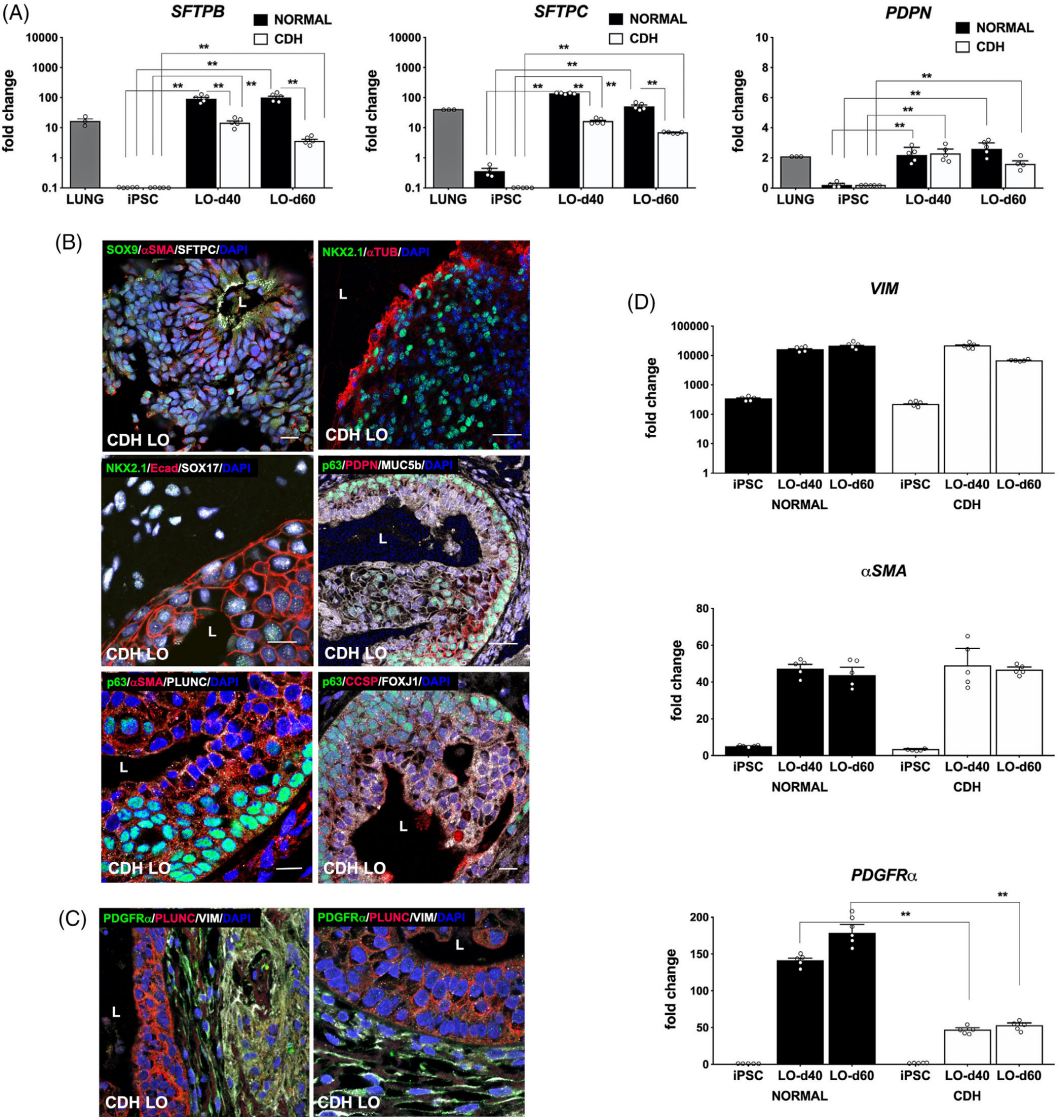
In the absence of mechanical compression, human lung organoids (LO) derived from induced pluripotent stem cells (iPSCs) of congenital diaphragmatic hernia (CDH) children showed differences in lung epithelial and mesenchymal cell differentiation. A, Vertical bar graphs of serial quantitative RT‐PCR confirmed the expression of distal lung epithelial‐associated genes in normal and CDH LOs, demonstrating upregulation compared to parental iPSCs but significantly decreased expression of type II alveolar epithelial cells, including surfactant protein‐B (*SFTPB*, *left*) and surfactant protein‐C (*SFTPC*, *middle*), in CDH LOs at both 40 and 60 days after LO induction. Expression of the type I alveolar epithelial cell marker, podoplanin (*PDPN*, *right*), was similar between LOs and CDH LOs in multiple time points. Data were normalized relative to housekeeping gene (*GAPDH*) and presented as the mean ± SEM, ** denotes *P* ≤ .01 (ANOVA and Mann‐Whitney, as appropriate), n = 4 independent biological replicates (three separate experiments using three different cell lines) without mechanical compression, and human fetal normal lung as a positive control. B, Representative confocal microscopy sections of human amniotic fluid‐derived CDH LOs (day 40) reveal multiple proximal and distal lung progenitor cell types within a surrounding mesenchyme. There are SOX9^+^SFTPC^+^ cells (FITC/TxRed secondary, green/red) lining a sac‐like structure within αSMA^+^ mesenchyme (*top row*, *left*; Cy5, white; magnification, ×40). “L” indicates luminal structure. Acetylated alpha‐tubulin (αTUB; Txred secondary, red) positive staining of ciliary epithelium adjacent to NKX2.1^+^ cells (FITC, green; *top row*, *right*; magnification, ×20). High‐power image demonstrates proximal airway‐like stratified epithelium E‐cadherin (Ecad)^+^ (TxRed, red) with continued expression of NKX2.1^+^ progenitors and SOX17^+^ cells (*second row*, *left*; FITC/Cy5, green/white); magnification, ×60). There are p63^+^ (bronchial reserve) cells (FITC, green) completely lining the outside of sac‐like structures within a background of plate‐like PDPN^+^ and more rounded MUC5b^+^ goblet cells (*second row*, *right*; TxRed/Cy5, red/white; magnification, ×20). Robust staining of α‐smooth muscle Actin (αSMA) and secretory PLUNC (TxRed/Cy5, red/white) near p63^+^ cells (FITC, green), suggestive of proximal lung epithelium (*third row*, *left*; magnification, ×60). The presence of multiple proximal airway cell types, as shown by club cell secretory protein (CCSP) and FOXJ1 (TxRed/Cy5, red/white), is shown (*third row*, *right*; magnification, ×40). Scale bars represent 10 μm. “L” indicates the lumen. C, High‐power confocal images revealing well‐organized PDGFRα^+^/VIM^+^ cells (FITC/Cy5, green/white), indicative of myofibroblastic differentiation, along the periphery of a PLUNC^+^ luminal structure (TxRed, red; magnification, ×60). D, Vertical bar graphs with dot plots of serial quantitative mesenchymal gene expression in amniotic fluid‐derived normal and CDH LOs show upregulation of *VIM*, *αSMA*, and *PDGFRα* compared to parental iPSCs. Whereas expression of *VIM* (*top)* and *αSMA* (*middle*) were similar between LOs and CDH LOs, there was significantly decreased expression of *PDGFRα (bottom*) in CDH LOs at both day 40 and day 60. Data were normalized relative to housekeeping gene (*GAPDH*) and presented as the mean ± SEM, ** denotes *P* ≤ .01 compared to normal LOs at same induction time (Mann‐Whitney), two independent biological replicates without mechanical compression (three separate experiments using three different cell lines). ANOVA, analysis of variance; RT‐PCR, real‐time polymerase chain reaction

We further characterized specific pulmonary epithelial and mesenchymal cell phenotypes in day 40 and day 60 CDH LOs by serial quantitative RT‐PCR. We measured two type II lung epithelial markers, namely surfactant protein‐B (*SFTPB*) and *SFTPC*, and found significantly lower expression of both genes in CDH LOs (Figure 5A, *P* ≤ .01). HOPX and PDPN were also evaluated and were significantly elevated to comparable levels compared to parental iPSCs in both normal and CDH LOs.

Pulmonary differentiation of CDH LOs into specific epithelial subtypes was shown by confocal microscopy, displaying numerous proximal lung progenitor cell types by day 40 (Figure 5B). There were multiple proximal airway subtypes, as shown by positive acetylated alpha‐tubulin (ciliated), MUC5b (goblet), PLUNC (secretory), p63, and club cell secretory protein staining. Staining of the stromal compartment within CDH LOs revealed well‐organized PDGFRα^+^/VIM^+^ myofibroblasts along the periphery of lumen‐like structures (Figure 5C).

Serial quantitative gene expression data of normal and CDH LOs revealed upregulation of *VIM*, *αSMA*, and *PDGFRα* (Figure 5D). However, whereas levels of expression of *VIM* and *αSMA* were similar between normal and CDH LOs, there was significantly decreased expression of *PDGFRα* in CDH LOs tested at both day 40 and day 60 (*P* ≤ .01). Taken together, these results suggest possible differences in type II alveolar epithelial cell and PDGFRα‐related differentiation among CDH LOs in the absence of mechanical compression forces.

### 3.5 Ex vivo mechanical compression alters human CDH LO development

**FIGURE 6:**
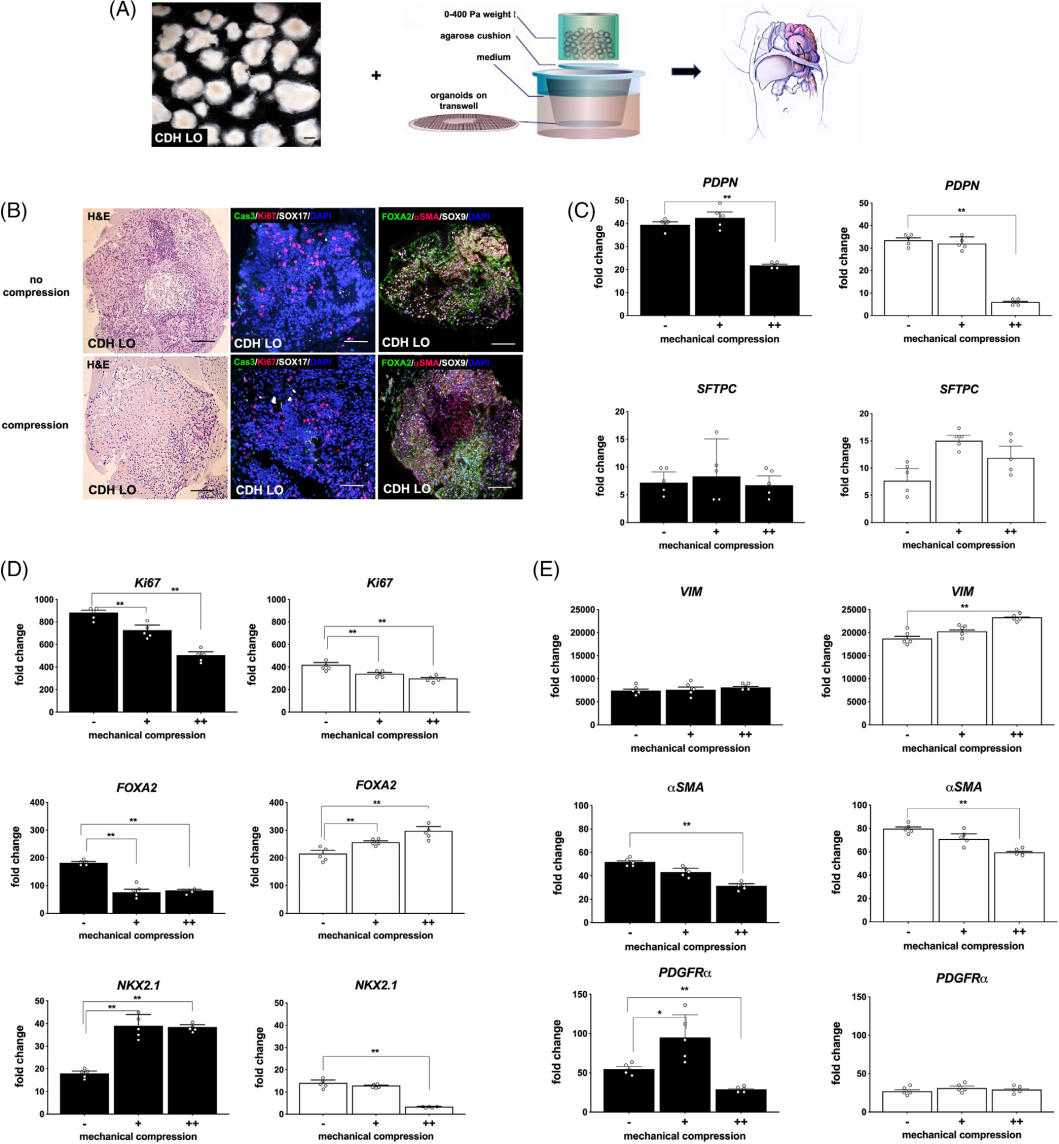
Ex vivo mechanical compression was associated with alterations in human congenital diaphragmatic hernia lung organoid (CDH LO) epithelial and mesenchymal gene regulation. A, Schematic representation of the compression device, in which day 40 CDH LOs (*left*) are exposed to static compression (*middle*, adapted from Reference 33) to simulate in utero forces (*right*). Scale bar represents 100 μm. B, Representative low‐power photomicrographs of CDH LOs under static mechanical compression (400 Pa) for 48 hours (*bottom row*). Controls consisting of CDH LOs derived from the same iPSC clones but without mechanical compression were cultured in parallel (*top row*). CDH LOs reveal no obvious differences with respect to H&E (*left panels*) after mechanical compression. Active Ki67/caspase‐3 (Cas3) immunofluorescent staining (*middle panels*; TxRed/FITC secondary, red/green) confirms ongoing cell proliferation and apoptosis in both groups. Confocal images illustrate SOX9 (Cy5 secondary, white) and α‐smooth muscle Actin (αSMA; TxRed secondary, red) distributed throughout the entire LO, regardless of mechanical compression (*right panels*). Scale bars represent 50 μm. C, Vertical bar graphs with dot plots show quantitative gene expression in normal LOs (black bars) and CDH LOs (white bars) under two different static mechanical compression forces (+ = 200 Pa vs ++ = 400 Pa) for 48 hours. Mechanical compression at 400 Pa was associated with significant downregulation of podoplanin (*PDPN*) but not surfactant protein‐C (*SFTPC*) when compared to noncompression controls (− = 0 Pa). Data were normalized relative to housekeeping gene (*GAPDH*) and presented as the mean ± SEM. ** denotes *P* ≤ .01 compared to the respective noncompressed LOs (Mann Whitney), independent biological replicates (n = 3 for CDH LOs, n = 2 for normal LOs). D, Higher mechanical compression was associated with significant downregulation in *Ki67* and *NKX2.1* but differential responses in *FOXA2*. ** denotes *P* ≤ .01 compared to the respective noncompressed LOs (ANOVA). E, Higher mechanical compression was associated with significant upregulation in vimentin (*VIM*) that was specific to CDH LOs but differential responses in PDGFRα. * and ** denote *P* ≤ .05 and *P* ≤ .01, respectively (ANOVA). iPSC, induced pluripotent stem cell. ANOVA, analysis of variance

To study the contribution that extrinsic compression may play in the development of the CDH lung phenotype, we used an ex vivo mechanical compression apparatus as previously described^33^, ^34^ and tested its effect on day 40 LOs that were otherwise cultured under the same growth conditions (Figure 6A). This time point was chosen since LOs differentiation was analogous to the pseudoglandular stage of lung development based on previous work.^31^, ^38^ In an effort to mimic in utero mechanical forces in fetal CDH, 100 Pa (1.4 cm H_2_O), 200 Pa (2.7 cm H_2_O), or 400 Pa (5.4 cm H_2_O) of static compression forces were used.

Although gross inspection was notable for obvious flattening of the organoid tissue after compression, confocal microscopy images showed no obvious microarchitectural differences by H&E staining (Figure 6B, left). Evidence of Ki67/Cas3 immunofluorescent staining suggested ongoing cell proliferation and apoptosis (Figure 6B, middle). There was significant gene downregulation in *PDPN* (at 400 Pa only) and *Ki67*, suggesting an overall reduction in organoid cell proliferation caused by the compression stimulus (Figure 6C, *P* ≤ .01). CDH‐specific alterations in gene expression under increased mechanical compression were demonstrated by significant reduction in *NKX2.1* (Figure 6D, *P* ≤ .01), and upregulation in *FOXA2* (Figure 6D, *P* ≤ .01) and *VIM* (Figure 6E, *P* ≤ .01) with escalating pressures. In contrast, there was specific and significant upregulation in *NKX2.1* in normal LOs with mechanical compression (Figure 6D, *P* ≤ .01).

**FIGURE 7:**
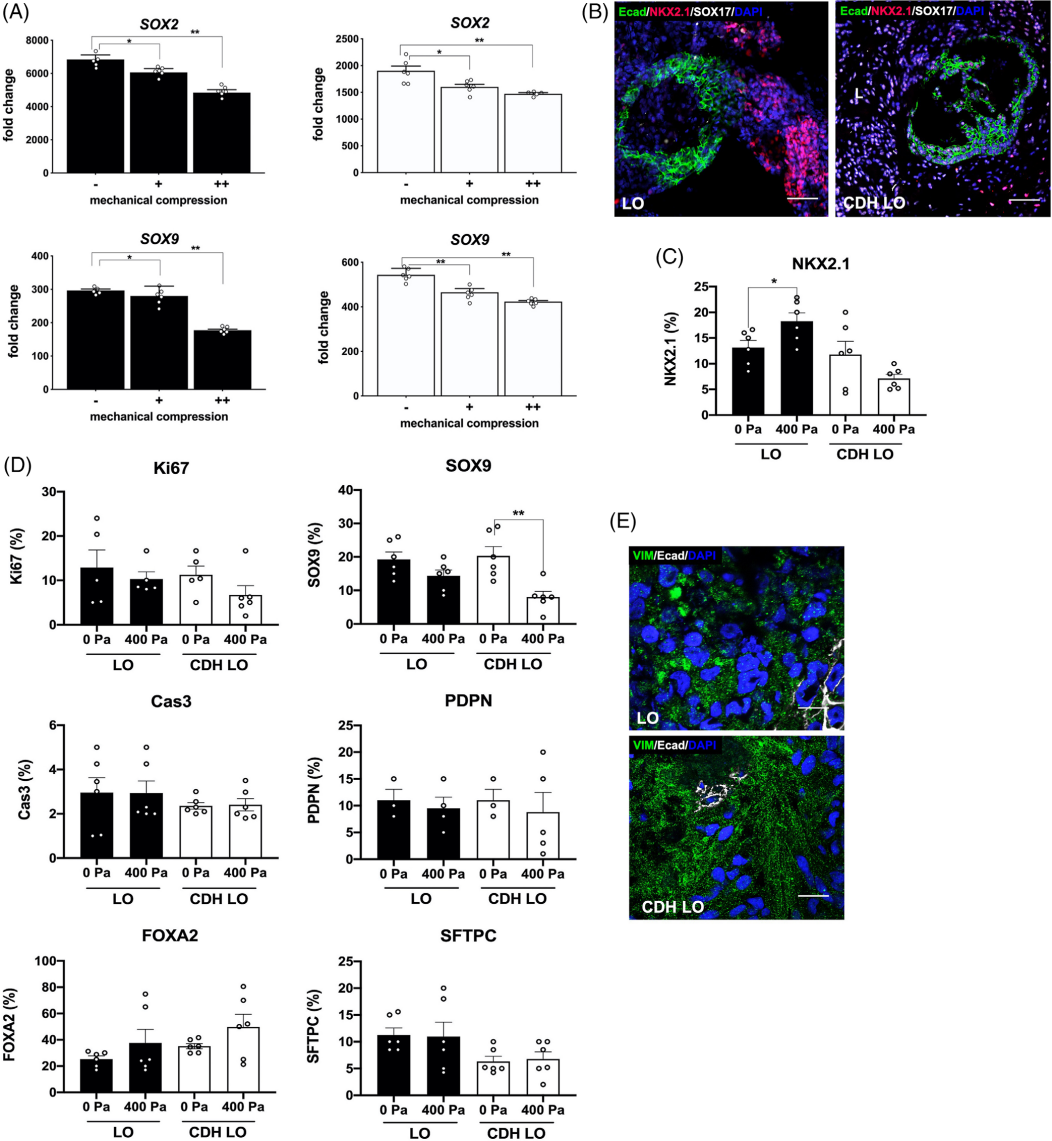
Further evidence that ex vivo mechanical compression altered human congenital diaphragmatic hernia lung organoid (CDH LO) epithelial and mesenchymal development. A, Vertical bar graphs with dot plots show significant downregulation of *SOX2* and *SOX9* gene expression in CDH and normal LOs under two different static mechanical compression forces (+ = 200 Pa vs ++ = 400 Pa) for 48 hours. Mechanical compression downregulates both *SOX2* and *SOX9* expression in a pressure‐dependent manner. Data were normalized relative to housekeeping gene (*GAPDH*) and presented as the mean ± SEM, * and ** denote *P* ≤ .05 and *P* ≤ .01, respectively, independent biological replicates (n = 3 for CDH LOs, n = 2 for normal LOs). B, Representative confocal microscopy sections (magnification, ×10) of LOs and CDH LOs after exposure to 200 Pa of mechanical compression, showing E‐cadherin (Ecad) and NKX2.1 protein expression (FITC and TxRed secondary, green/red). Scale bars represent 150 μm. C, Vertical bar graph with dot plots of NKX2.1 protein expression before and after mechanical compression reveals differential responses between LOs and CDH LOs. * denotes *P* ≤ .05 (Mann‐Whitney). D, Vertical bar graphs with dot plots of selected protein expression before and after mechanical compression show significant downregulation in SOX9. ** denotes *P* ≤ .01 (Mann‐Whitney). E, Representative confocal microscopy sections (magnification, ×60) after exposure to 400 Pa of mechanical compression, demonstrating E‐cadherin (Ecad) and robust vimentin (VIM) staining (Cy5 and FITC secondary, white/green) in CDH LOs. Scale bars represent 25 μm

In parallel with these findings, there was significant stepwise downregulation in the expression of lung progenitor genes, *SOX2* and *SOX9*, (Figure 7A) in both CDH and normal LOs at higher pressures (*P* ≤ .01). Increased NKX2.1 in response in mechanical compression among normal LOs was supported by immunofluorescence (Figure 7B) and shown by quantitative protein expression (Figure 7C). Compression was associated with reduced SOX9 protein expression that was specific to CDH LOs (Figure 7D). Confocal images revealed evidence for increased VIM expression within the cytoplasm of CDH LOs (Figure 7E), although these levels could not be formally quantified. Combined, these data lend support to the concept of disease‐ and cell phenotype‐specific changes in response to mechanical compression in CDH, resulting in altered progenitor, epithelial, and mesenchymal cell development within the developing lung.

## 4 DISCUSSION

CDH is a perplexing congenital lung anomaly whose exact cause remains unknown.^39^ Although somatic mutations in *GATA4 and FOG2*, among others, have been implicated in less than 10% of CDH patients,^15^, ^16^, ^40^ the etiology of CDH is heterogeneous and likely polygenic in the majority of cases.^12^ Regardless, the downstream result of the near‐term CDH fetal lung is progressive hypoplasia involving both proximal and distal airways as well as abnormalities within the adjacent mesenchyme and airway smooth muscle.^21^, ^41^ At autopsy in both CDH fetuses and infants, pathologic evaluation of the lung uniformly reveals a spectrum of developmental abnormalities, including impairments in alveolarization, decreased vascularization, and smooth muscle arteriole hypertrophy.^41^, ^42^, ^43^ The ability to elucidate the mechanisms of disease contributing to these lung derangements has been mixed despite decades of animal model research.^44^, ^45^

In this study, we report on the directed differentiation of human iPSCs derived from CDH amniotic fluid and neonatal skin into 3D fetal lung‐like structures using an established and highly reproducible protocol.^32^ In addition to previous work demonstrating the presence of gene, protein, and electron microscopy data on distal lung differentiation, bulk RNA‐seq analyses have shown that organoids from non‐CDH patients produced by this method are more similar to native fetal lung than to adult lung.^31^ We then subjected our CDH LOs to ex vivo mechanical forces to evaluate the contribution of pathologic compression on pulmonary development. Although the role of mechanical strain on lung epithelia in 2D culture has been studied by others to understand mechanisms of ventilator‐associated lung injury and other acquired pathologies,^46^, ^47^ the role of mechanical factors on multicellular human organoids has not been explored in an ex vivo setting. Here, by simulating what would occur when the CDH fetal lung becomes physically constrained by space‐occupying abdominal organs within the pleural space, we observed a reproducible and stepwise downregulation in the expression of lung progenitor genes, *SOX2* and *SOX9*, with increasing mechanical pressures (Figure 7). There was also a profound decrease in *NKX2.1* and *PDPN* gene expression along with an increase in *FOXA2* and *VIM* gene expression in CDH LOs (Figure 6), reflecting the differential effects of mechanical forces on distinct cell types within developing organoids.

Lack of NKX2.1^+^ cells within the developing lung has been associated with loss of type I pneumocytes and perinatal tissue damage,^48^, ^49^ whereas upregulation of *FOXA2* has been shown to correlate with proximal airway epithelial cell and type II alveolar proliferation.^50^ The negative effect of mechanical compression on *PDPN*, a gene expressed in gas‐exchanging (type I) pneumocytes, is not trivial since these cells cover 95% of the alveolar surface of the lung^51^ and was recently confirmed to be markedly diminished in a murine model of CDH.^52^ In contrast, type II pneumocytes serve as alveolar stem cells and secrete pulmonary surfactant.^53^ The increased expression of *VIM* is consistent with a pro‐fibroblastic response in setting of an nonphysiological mechanical stimulus.^54^ Taken together, since we were able to quantitatively apply disease relevant compression forces to our CDH LOs roughly corresponding to the pseudoglandular and early canalicular stages of development, these findings suggest a new in vitro research platform to study the mechanobiology and patient‐specific disease pathogenesis of human CDH fetal lung hypoplasia.

Although mechanical pressure gradients play an important and essential role in regulating normal lung morphogenesis,^55^, ^56^, ^57^ the role of *NKX2.1* in normal and CDH lung development remains relatively unknown.^58^ The fetus itself is known to initiate “breathing” movements, where amniotic fluid is intermittently inhaled and exhaled starting during the canalicular stage of pulmonary development.^59^, ^60^, ^61^ It has also been shown that there are spontaneous peristaltic airway contractions within the fetal lung that can propel lung liquid through the bronchial tree.^62^ Using embryonic murine lung explants within bioengineered microfluidic devices, increased transmural pressures can accelerate the rate of airway epithelial morphogenesis due to crosstalk between different tissues.^17^ The strategy to induce lung growth in CDH through the augmentation of transmural (intrapulmonary) pressures has already been applied experimentally in humans by fetoscopic tracheal occlusion (FETO), a procedure performed at our hospital and other selected fetal care centers worldwide.^4^, ^63^ During FETO‐induced lung growth in experimental models, a marked proliferation of type I alveolar epithelial cells, potentially involving the FGF10‐FGFR2b‐Sprouty2 pathway, has been shown.^64^, ^65^ FETO does not appear to increase NKX2.1 expression in either normal or CDH fetal lungs in late gestational animal models.^64^, ^66^

Despite the lack of contiguous airway branching structures within our 3D LOs, we mimicked the low intrapulmonary transmural pressures seen clinically in CDH by applying external mechanical forces in an ex vivo system. We found an overall reduction in cell proliferation using *Ki67* (Figure 5A) as well as impaired expression of *NKX2.1* and *PDPN* (Figure 5D). Our data are also concordant with previous work based on our ex vivo compression apparatus used to assess changes in fetal rat lungs.^34^ In that study, we found that mechanical compression impaired normal lung growth based on branching morphometric analyses but did not significantly alter surfactant protein or *αSMA* gene expression.

Perhaps the most intriguing observation in our study is the data suggesting an inherent impairment in LO development in CDH patients, even in the absence of mechanical compression forces. Compared with LOs derived from normal, non‐CDH patients cultured under identical conditions, the differentiation of CDH LOs was associated with impaired *NKX2.1*, *SOX2*, *SOX9*, *SFTPB*, *SFTPC*, and *PDGFRα* expression under routine conditions. PDGFRα, a tyrosine kinase receptor expressed by alveolar myofibroblasts, has been shown to be critical for early lung branching and alveolarization.^67^, ^68^ Approximately 10% of ECM‐related genes, many associated with proximal/bronchial airway formation, were significantly upregulated in day 40 CDH LOs in microarray analyses (Figure 3D). Collectively, these findings support the concept of CDH as a global embryopathy,^69^ which argues that a more basic, mesenchymal cell‐intrinsic disturbance during early fetal development may exist in these patients, leading to pleiotropic effects on the lung and adjacent diaphragm.^12^, ^21^, ^70^ In fetal rodent models of CDH lung hypoplasia, global embryopathy as a cause of CDH has been supported by observations of abnormal branching morphogenesis that precedes normal formation of the diaphragm as early as E11.^25^, ^71^ Moreover, there is always lung hypoplasia in these models despite the fact that approximately half of fetal pups never develop the diaphragmatic hernia itself.^72^ Although experimental work in our laboratory^73^ has also shown that hypoplastic fetal lungs in the nitrofen rat model of CDH undergo some catch‐up lung growth when explanted and cultured ex vivo in the absence of compression forces, others have observed persistent hypoplasia under similar conditions.^74^

Given the findings in our study, the application of mechanical compression may represent a “second hit” in which lung hypoplasia is further aggravated, perhaps by aberrant mesenchymal‐epithelial responses to compression stimuli as we and others have previously suggested in rodent models.^22^, ^34^ Some experimental evidence supports an underlying dysfunction in mesenchymal cells associated with defective alveolarization in CDH.^75^, ^76^, ^77^ Investigators have also shown that soluble factors secreted by the mesenchyme can have effects on alveolar progenitor cell differentiation.^78^ Others have found that PDGFRα‐expressing cells within the mesenchyme migrate to the tips of secondary alveolar septa and differentiate into α‐actin‐ and elastin‐producing myofibroblasts that are required for normal alveolar development and gas exchange.^67^, ^68^, ^79^ We speculate that PDGFRα^+^ cells in CDH may be impaired in many patients, resulting in subsequent downstream effects on early lung branching and alveolarization.^80^ The lack of significant changes in *αSMA* gene expression, a general marker of smooth muscle cells, among CDH LOs when compared to control LOs is discordant with nitrofen rat studies^81^ but is consistent with recent autopsy reports conducted in term CDH infants.^82^

Reduced expression of *SHH* during the pseudoglandular phase of lung development has been implicated in the nitrofen model.^83^ Our microarray data also revealed decreased *SHH* expression in day 40 CDH LOs, but this narrowly missed statistical significance. Although we found evidence for increased ECM gene expression in CDH LOs (Figure 3G), evaluation using RNA‐seq technology would be instrumental toward gaining a better understanding of the genes and molecular regulatory networks involved in mediating early lung development with or without the presence of mechanical forces. Previous studies on transcriptome profiling of human fetal lungs are now readily available.^38^ The role of *FOXA2* in CDH and whether patients with a more severe CDH phenotype have more intrinsic derangements during LO differentiation remain unknown and are currently under investigation in our laboratory.

Despite the promising data presented in this study, there are notable limitations to this work. First, the creation of 3D LOs from human iPSCs requires months and remains labor intensive. As a result, we studied the effect of one type of mechanical compression at one specific time point in CDH organoid differentiation. It is likely that the application of various types of mechanical forces, such as intermittent compression or chronic compression, may induce unique and different time‐dependent effects on organoid differentiation.^17^, ^84^ Second, further work is needed using additional CDH and normal control samples given that CDH is a polygenic disorder across a wide disease spectrum, and there are likely to be additional variations in the differentiation capabilities of various iPSC clones, which is beyond the scope of our study. There are more than 20 different genetic mutations that have been described in nonsyndromic CDH, and a laboratory gene panel does not currently exist to screen for these genes.^10^ Third, it is possible that alternative directed differentiation protocols, as we and others have described,^85^, ^86^, ^87^ may yield different results within our ex vivo compression system. Fourth, unlike hypoplastic fetal rodent lung explants used in our laboratory and elsewhere,^73^ this organoid model is not suitable for studying aberrant CDH branching morphogenesis^86^ and the subsequent instructional role that airway smooth muscle may play during this process.^88^ Finally, it is difficult to evaluate function in LOs, and we cannot evaluate morphometric aspects of LOs, such size and complexity, as well as other components of lung development including pulmonary blood vessel formation, an important aspect in CDH lung development.^89^, ^90^ Studies of the latter would likely require the in vivo transfer of LOs as our laboratory and others have previously described using LOs derived from non‐CDH patients.^91^, ^92^

## 5 CONCLUSION

In this proof of concept paper, we describe a stem cell‐based approach to facilitate the study of disease pathogenesis in CDH fetal lung hypoplasia. Ongoing studies using these CDH organoids should allow us to better understand epithelial‐mesenchymal cell interactions that occur early in this unique disease‐specific microenvironment.^78^ Further advancement of this translational work will also represent the necessary steps toward developing cell‐based therapies and the testing of experimental pharmacologic agents, including pulmonary morphogens and growth factors,^93^, ^94^, ^95^ as potential approaches to enhance perinatal lung development and differentiation in these fragile children.

## CONFLICT OF INTEREST

The authors declared no potential conflicts of interest.

## AUTHOR CONTRIBUTIONS

S.M.K.: conception and design, financial support, provision of study material/patients, data analysis and interpretation, manuscript writing; G.J.: conception and design, collection and assembly of data, data analysis and interpretation, manuscript writing, final approval of manuscript; J.C.B.: collection and assembly of data, data analysis and interpretation, manuscript writing, final approval of manuscript; K.K.Y.H., B.R.D.: collection and assembly of data, data analysis and interpretation, final approval of manuscript; A.P.L.: conception and design, data analysis and interpretation, final approval of manuscript; J.R.S.: conception and design, financial support, data analysis and interpretation, final approval of manuscript.

## Supporting information


**Appendix** **S1.** Supporting Information.

**Figure S1** Generation of human pluripotent stem cells derived from congenital diaphragmatic hernia (CDH) patients. **(A)** Representative phase contrast photomicrographs of foreskin and amniotic fluid mesenchymal cells at passage 4 (*upper panels*, magnification, 60x) prior to reprogramming. Representative colony morphology of induced pluripotent stem cells (iPSCs) at 10x (*middle panels*) and 40x (*lower panels*) magnification after exposure to Sendai virus. **(B)** Representative alkaline phosphatase staining and immunofluorescence profile of CDH iPSCs shows marked similarities with iPSCs from children with normal lungs as well as human embryonic stem cell (ESC) controls (magnification, 20x). Scale bars represent 100 μm. **(C)** Representative karyotype analysis of iPSC colonies (passage 10) derived from neonatal foreskin shows normal chromosomes based on twenty 20 G‐banded metaphase cells. **(D)** Immunofluorescence microscopy of representative adherent embryoid bodies (EB) from normal and CDH iPSCs demonstrates similar spontaneous three germ layer expression of β‐III tubulin (ectoderm), SOX17 (endoderm), and αSMA (mesoderm) superimposed with DAPI, magnification, 40x. Scale bars represent 50 μm.

**Figure S2** Characterization of additional clones derived from human pluripotent stem cells from congenital diaphragmatic hernia (CDH) patients. **(A)** Representative phase contrast photomicrographs of colony morphology of induced pluripotent stem cells (iPSCs) at 10x (*top panels*) after exposure to Sendai virus. **(B)** Representative alkaline phosphatase staining and immunofluorescence profile of CDH iPSCs shows marked similarities with iPSCs from children with normal lungs as well as human embryonic stem cell (ESC) controls (magnification, 20x). Scale bars represent 100 μm. **(C)** Vertical bar graphs with dot plots demonstrate significant upregulation of pluripotency‐specific genes, including *NANOG*, *OCT4*, *and SOX2*, in iPSC clones (shown in white) derived from four different CDH patients (CDH1‐CDH4, passage 22‐26) compared to that in respective parental cell controls (passage 4). Data were normalized relative to housekeeping gene (*GAPDH*) and presented as the mean ± SEM, ** denotes *P* ≤ 0.01 compared to control (Mann‐Whitney). **(D)** Representative karyotype analysis of iPSCs colonies (passages 8‐14) derived from neonatal foreskin or amniotic fluid shows normal chromosomes based on twenty 20 G‐banded metaphase cells. **(E)** Immunofluorescence microscopy of representative adherent embryoid bodies (EB) from normal and CDH iPSCs demonstrates similar spontaneous three germ layer expression of β‐III tubulin (ectoderm), SOX17 (endoderm), and αSMA (mesoderm) superimposed with DAPI (magnification, 40x). Scale bars represent 50 μm.

## Data Availability

The data used to support the findings of this study are available from the corresponding author upon request.
