# Supplementary material for: Human induced pluripotent stem cell‐derived lung organoids in an ex vivo model of the congenital diaphragmatic hernia fetal lung
Source: Stem Cells Transl Med. 2020 Sep 19;10(1):98–114. doi: 10.1002/sctm.20-0199 (PMC7780804; doi:10.1002/sctm.20-0199)
Supplement: Supplementary file 1 — Appendix S1. Supporting Information. [file SCT3-10-98-s001.docx]

**SUPPLEMENTAL INFORMATION**

**Human induced pluripotent stem cell-derived lung organoids in an *ex vivo* model of the congenital diaphragmatic hernia fetal lung**

Shaun M. Kunisaki, MD, MSc^1,2^; Guihua Jiang, MS^3^; Juan C. Biancotti, PhD^1,2^; Kenneth K.Y. Ho, BE^4^; Briana R. Dye, PhD^5^; Allen P. Liu, PhD^4^; and Jason R. Spence, PhD^5,6^

**Supplemental Figure Legends**

**Figure S1:** Generation of human pluripotent stem cells derived from congenital diaphragmatic hernia (CDH) patients. **(A)** Representative phase contrast photomicrographs of foreskin and amniotic fluid mesenchymal cells at passage 4 (*upper panels*, magnification, 60x) prior to reprogramming. Representative colony morphology of induced pluripotent stem cells (iPSCs) at 10x (*middle panels*) and 40x (*lower panels*) magnification after exposure to Sendai virus. **(B)** Representative alkaline phosphatase staining and immunofluorescence profile of CDH iPSCs shows marked similarities with iPSCs from children with normal lungs as well as human embryonic stem cell (ESC) controls (magnification, 20x). Scale bars represent 100 μm. **(C)** Representative karyotype analysis of iPSC colonies (passage 10) derived from neonatal foreskin shows normal chromosomes based on twenty 20 G-banded metaphase cells. **(D)** Immunofluorescence microscopy of representative adherent embryoid bodies (EB) from normal and CDH iPSCs demonstrates similar spontaneous three germ layer expression of β-III tubulin (ectoderm), SOX17 (endoderm), and αSMA (mesoderm) superimposed with DAPI, magnification, 40x. Scale bars represent 50 μm.

**Figure S2:** Characterization of additional clones derived from human pluripotent stem cells from congenital diaphragmatic hernia (CDH) patients. **(A)** Representative phase contrast photomicrographs of colony morphology of induced pluripotent stem cells (iPSCs) at 10x (*top panels*) after exposure to Sendai virus. **(B)** Representative alkaline phosphatase staining and immunofluorescence profile of CDH iPSCs shows marked similarities with iPSCs from children with normal lungs as well as human embryonic stem cell (ESC) controls (magnification, 20x). Scale bars represent 100 μm.

**(C)** Vertical bar graphs with dot plots demonstrate significant upregulation of pluripotency-specific genes, including *NANOG, OCT4, and SOX2*, in iPSC clones (shown in white) derived from four different CDH patients (CDH1-CDH4, passage 22-26) compared to that in respective parental cell controls (passage 4). Data were normalized relative to housekeeping gene (*GAPDH*) and presented as the mean±SEM, ** denotes p≤0.01 compared to control (Mann-Whitney). **(D)** Representative karyotype analysis of iPSCs colonies (passages 8-14) derived from neonatal foreskin or amniotic fluid shows normal chromosomes based on twenty 20 G-banded metaphase cells. **(E)** Immunofluorescence microscopy of representative adherent embryoid bodies (EB) from normal and CDH iPSCs demonstrates similar spontaneous three germ layer expression of β-III tubulin (ectoderm), SOX17 (endoderm), and αSMA (mesoderm) superimposed with DAPI (magnification, 40x). Scale bars represent 50 μm.

**Table S1.** Clinical characteristics of congenital diaphragmatic hernia (CDH) patients

| *Patient* | *Gender* | *Cell source* | *Age at collection* | *Other anomalies* | *Family history* | *CDH*  *side* | *Disease phenotype* |
| --- | --- | --- | --- | --- | --- | --- | --- |
| CDH1 | Male | Foreskin | 5 weeks | None | None | Left | Mild |
| CDH2 | Female | Amniotic fluid | 18 weeks* | None | None | Left | Moderate |
| CDH3 | Male | Foreskin | 5 weeks | None | None | Left | Severe |
| CDH4 | Male | Foreskin | 3 weeks | Small ASD | None | Left | Mild |
| CDH5 | Male | Amniotic fluid | 24 weeks* | None | None | Left | Severe |
| CDH6 | Male | Foreskin | 8 weeks | None | None | Left | Mild |

Abbreviations: ASD, atrial septal defect

* denotes gestational age

**Table S2.** Lung development genes in microarray panel

| **Symbol** | **Name** |  |
| --- | --- | --- |
| AARD | alanine and arginine rich domain containing protein | |
| ACVR2B | activin A receptor type 2B | |
| ADAMTS2 | ADAM metallopeptidase with thrombospondin type 1 motif 2 | |
| AGER | advanced glycosylation end product-specific receptor | |
| CCDC40 | coiled-coil domain containing 40 | |
| CHI3L1 | chitinase 3 like 1 | |
| CP | ceruloplasmin (ferroxidase) | |
| CRISPLD2 | cysteine rich secretory protein LCCL domain containing 2 | |
| CTGF | connective tissue growth factor | |
| DHCR7 | 7-dehydrocholesterol reductase | |
| DICER1 | dicer 1 ribonuclease III | |
| DNAAF1 | dynein (axonemal) assembly factor 1 | |
| EIF4EBP1 | eukaryotic translation initiation factor 4E binding protein 1 | |
| EPAS1 | endothelial PAS domain protein 1 | |
| FBXW7 | F-box and WD repeat domain containing 7 | |
| FGF1 | fibroblast growth factor 1 | |
| FGF18 | fibroblast growth factor 18 | |
| FGFR2 | fibroblast growth factor receptor 2 | |
| FOXF1 | forkhead box F1 | |
| FSTL3 | follistatin like 3 | |
| GLI1 | GLI family zinc finger 1 | |
| GLI2 | GLI family zinc finger 2 | |
| GLI3 | GLI family zinc finger 3 | |
| GPC3 | glypican 3 | |
| HEG1 | heart development protein with EGF like domains 1 | |
| HES1 | hes family bHLH transcription factor 1 | |
| HMGCS2 | 3-hydroxy-3-methylglutaryl-CoA synthase 2 | |
| HSD11B1 | hydroxysteroid (11-beta) dehydrogenase 1 | |
| ITGA3 | integrin subunit alpha 3 | |
| JMJD6 | jumonji domain containing 6 | |
| LAMA5 | laminin subunit alpha 5 | |
| LOX | lysyl oxidase | |
| MMP14 | matrix metallopeptidase 14 | |
| NOTCH1 | notch 1 | |
| PDPN | podoplanin | |
| PPP1CA | protein phosphatase 1 catalytic subunit alpha | |
| PROX1 | prospero homeobox 1 | |
| PTN | pleiotrophin | |
| RAB3A | RAB3A, member RAS oncogene family | |
| RBP4 | retinol binding protein 4 | |
| RIDA | reactive intermediate imine deaminase A homolog | |
| SHH | sonic hedgehog | |
| SIM2 | single-minded family bHLH transcription factor 2 | |
| SPARC | secreted protein acidic and cysteine rich | |
| SREBF1 | sterol regulatory element binding transcription factor 1 | |
| STRA6 | stimulated by retinoic acid 6 | |
| TBX4 | T-box 4 | |
| TBX5 | T-box 5 | |
| TIMELESS | timeless circadian clock | |
| VEGFA | vascular endothelial growth factor A | |
| WNT2 | wingless-type MMTV integration site family member 2 | |
| WNT7B | wingless-type MMTV integration site family member 7B | |
| ZIC3 | Zic family member 3 | |

**Table S3.** Extracellular matrix genes in microarray panel

| **Symbol** | **Name** |  |
| --- | --- | --- |
| ABI3BP | ABI family member 3 binding protein | |
| ACAN | aggrecan | |
| ADAM11 | ADAM metallopeptidase domain 11 | |
| ADAMTS10 | ADAM metallopeptidase with thrombospondin type 1 motif 10 | |
| ADAMTS12 | ADAM metallopeptidase with thrombospondin type 1 motif 12 | |
| ADAMTS20 | ADAM metallopeptidase with thrombospondin type 1 motif 20 | |
| ADAMTS3 | ADAM metallopeptidase with thrombospondin type 1 motif 3 | |
| ADAMTS4 | ADAM metallopeptidase with thrombospondin type 1 motif 4 | |
| ADAMTS8 | ADAM metallopeptidase with thrombospondin type 1 motif 8 | |
| ADAMTS9 | ADAM metallopeptidase with thrombospondin type 1 motif 9 | |
| AGRN | agrin | |
| AMELY | amelogenin, Y-linked | |
| APOE | apolipoprotein E | |
| ASPN | asporin | |
| B4GALT7 | xylosylprotein beta 1,4-galactosyltransferase, polypeptide 7 | |
| BGN | biglycan | |
| BMP7 | bone morphogenetic protein 7 | |
| CDON | cell adhesion associated, oncogene regulated | |
| CFP | complement factor properdin | |
| CILP | cartilage intermediate layer protein | |
| CLU | clusterin | |
| COCH | cochlin | |
| COL12A1 | collagen type XII alpha 1 | |
| COL14A1 | collagen type XIV alpha 1 | |
| COL15A1 | collagen type XV alpha 1 | |
| COL18A1 | collagen type XVIII alpha 1 | |
| COL1A1 | collagen type I alpha 1 | |
| COL1A2 | collagen type I alpha 2 | |
| COL21A1 | collagen type XXI alpha 1 | |
| COL26A1 | collagen type XXVI alpha 1 | |
| COL2A1 | collagen type II alpha 1 | |
| COL3A1 | collagen type III alpha 1 | |
| COL4A1 | collagen type IV alpha 1 | |
| COL4A2 | collagen type IV alpha 2 | |
| COL5A1 | collagen type V alpha 1 | |
| COL5A2 | collagen type V alpha 2 | |
| COL6A1 | collagen type VI alpha 1 | |
| COL6A2 | collagen type VI alpha 2 | |
| COL6A3 | collagen type VI alpha 3 | |
| COL7A1 | collagen type VII alpha 1 | |
| COL8A1 | collagen type VIII alpha 1 | |
| COL8A2 | collagen type VIII alpha 2 | |
| COMP | cartilage oligomeric matrix protein | |
| CPXM2 | carboxypeptidase X (M14 family), member 2 | |
| CRIP2 | cysteine rich protein 2 | |
| CTSD | cathepsin D | |
| CYR61 | cysteine rich angiogenic inducer 61 | |
| DCN | decorin | |
| DPT | dermatopontin | |
| ECM1 | extracellular matrix protein 1 | |
| EFEMP1 | EGF containing fibulin-like extracellular matrix protein 1 | |
| EGFL7 | EGF like domain multiple 7 | |
| EMILIN1 | elastin microfibril interfacer 1 | |
| EMILIN2 | elastin microfibril interfacer 2 | |
| EMILIN3 | elastin microfibril interfacer 3 | |
| F3 | coagulation factor III, tissue factor | |
| FBLN1 | fibulin 1 | |
| FBLN2 | fibulin 2 | |
| FBLN5 | fibulin 5 | |
| FBN1 | fibrillin 1 | |
| FBN2 | fibrillin 2 | |
| FBN3 | fibrillin 3 | |
| FGF10 | fibroblast growth factor 10 | |
| FGFBP3 | fibroblast growth factor binding protein 3 | |
| FGFR2 | fibroblast growth factor receptor 2 | |
| FMOD | fibromodulin | |
| FN1 | fibronectin 1 | |
| FRAS1 | Fraser extracellular matrix complex subunit 1 | |
| FREM3 | FRAS1 related extracellular matrix 3 | |
| HAPLN1 | hyaluronan and proteoglycan link protein 1 | |
| HMCN1 | hemicentin 1 | |
| HSPG2 | heparan sulfate proteoglycan 2 | |
| HTRA1 | HtrA serine peptidase 1 | |
| IGFBP7 | insulin like growth factor binding protein 7 | |
| ILK | integrin linked kinase | |
| KRT1 | keratin 1 | |
| LAMA1 | laminin subunit alpha 1 | |
| LAMA2 | laminin subunit alpha 2 | |
| LAMA4 | laminin subunit alpha 4 | |
| LAMA5 | laminin subunit alpha 5 | |
| LAMB1 | laminin subunit beta 1 | |
| LAMB2 | laminin subunit beta 2 | |
| LAMC1 | laminin subunit gamma 1 | |
| LGALS1 | lectin, galactoside-binding, soluble, 1 | |
| LGALS3 | lectin, galactoside-binding, soluble, 3 | |
| LGALS3BP | lectin, galactoside-binding, soluble, 3 binding protein | |
| LMCD1 | LIM and cysteine rich domains 1 | |
| LOXL1 | lysyl oxidase like 1 | |
| LPL | lipoprotein lipase | |
| LTBP1 | latent transforming growth factor beta binding protein 1 | |
| LTBP2 | latent transforming growth factor beta binding protein 2 | |
| LTBP3 | latent transforming growth factor beta binding protein 3 | |
| LTBP4 | latent transforming growth factor beta binding protein 4 | |
| LUM | lumican | |
| MATN2 | matrilin 2 | |
| MFAP4 | microfibrillar associated protein 4 | |
| MFGE8 | milk fat globule-EGF factor 8 protein | |
| MGP | matrix Gla protein | |
| MMP14 | matrix metallopeptidase 14 | |
| MMP15 | matrix metallopeptidase 15 | |
| MMP27 | matrix metallopeptidase 27 | |
| NDNF | neuron-derived neurotrophic factor | |
| NID1 | nidogen 1 | |
| NID2 | nidogen 2 | |
| OGN | osteoglycin | |
| PCOLCE | procollagen C-endopeptidase enhancer | |
| PCSK6 | proprotein convertase subtilisin/kexin type 6 | |
| PLSCR1 | phospholipid scramblase 1 | |
| POSTN | periostin, osteoblast specific factor | |
| PRELP | proline/arginine-rich end leucine-rich repeat protein | |
| PRSS2 | protease, serine 2 | |
| PRTN3 | proteinase 3 | |
| PXDN | peroxidasin | |
| RARRES2 | retinoic acid receptor responder (tazarotene induced) 2 | |
| SBSPON | somatomedin B and thrombospondin type 1 domain containing | |
| SERPINE1 | serpin peptidase inhibitor, clade E, member 1 | |
| SERPINE2 | serpin peptidase inhibitor, clade E, member 2 | |
| SERPINF1 | serpin peptidase inhibitor, clade F , member 1 | |
| SFRP1 | secreted frizzled-related protein 1 | |
| SFRP2 | secreted frizzled-related protein 2 | |
| SLPI | secretory leukocyte peptidase inhibitor | |
| SOD3 | superoxide dismutase 3, extracellular | |
| SPOCK3 | sparc/osteonectin, cwcv and kazal-like domains proteoglycan 3 | |
| SPON1 | spondin 1 | |
| SSC5D | scavenger receptor cysteine rich family, 5 domains | |
| TGFB1I1 | transforming growth factor beta 1 induced transcript 1 | |
| TGFB2 | transforming growth factor beta 2 | |
| TGFB3 | transforming growth factor beta 3 | |
| TGFBI | transforming growth factor beta induced | |
| THBS1 | thrombospondin 1 | |
| THBS4 | thrombospondin 4 | |
| TIMP3 | TIMP metallopeptidase inhibitor 3 | |
| TINAGL1 | tubulointerstitial nephritis antigen like 1 | |
| TNC | tenascin C | |
| TNXB | tenascin XB | |
| WNT2 | wingless-type MMTV integration site family member 2 | |
| ZP3 | zona pellucida glycoprotein 3 (sperm receptor) | |

**Table S4.** Antibody Information

| **Primary** | | **Clone** | | **Dilution** | | | **Source** | | | **Cat#** | | |  |
| --- | --- | --- | --- | --- | --- | --- | --- | --- | --- | --- | --- | --- | --- |
| Alpha smooth muscle actin (αSMA) | | IgG2a | | 1:1000 | | | Sigma | | | C6198 | | |  |
| Alpha tubulin | | Sigma | | 1:1000 | | | Sigma | | | T7451 | | |  |
| Cas3 | | polyclonal | | 1:200 | | | Cell Signaling | | | #9662 | | |  |
| E-cadherin | | IgG2a | | 1:500 | | | BD | | | 610181 | | |  |
| FOXA2 | | polyclonal | | 1:200 | | | Sevenhills | | | WRAB-1200 | | |  |
| FOXJ1 | | 2A5 | | 1:500 | | | eBioscience | | | 14-9965-82 | | |  |
| Ki67 | | monoclonal | | 1:200 | | | Millipore | | | MAB4190 | | |  |
| NKX2.1 | | monoclonal | | 1:200 | | | Abcam | | | ab76013 | | |  |
| p63 | | H-129 | | 1:200 | | | Santa Cruz | | | Sc-8344 | | |  |
| PAX8 | | polyclonal | | 1:200 | | | proteinteck | | | 10336-1 | | |  |
| PDGRF-alpha | | C-20 | | 1:100 | | | Santa Cruz | | | sc-338 | | |  |
| PDPN | | polyclonal | | 1:200 | | | Santa Cruz | | | sc-134482 | | |  |
| SFTPB | | polyclonal | | 1:200 | | | Santa Cruz | | | sc-13978 | | |  |
| SFTPC | | polyclonal | | 1:200 | | | Sevenhills | | | WRAB-9337 | | |  |
| SOX2 | | polyclonal | | 1:200 | | | Sevenhills | | | WRAB-1236 | | |  |
| SOX9 | | polyclonal | | 1:500 | | | R&D | | | AF3075 | | |  |
| SOX17 | | polyclonal | | 1:500 | | | R&D | | | AF1924 | | |  |
| Vimentin | | polyclonal | | 1:500 | | | Santa Cruz | | | sc-7558 | | |  |
|  |  | |  | | |  | | |  | | |  |  |
| **Secondary** |  | |  | | |  | | |  | | |  |  |
| Donkey anti-goat 488 |  | | 1:500 | | | Jackson | | | 705-545-147 | | |  |  |
| Donkey anti-goat AF647 |  | | 1:500 | | | Jackson | | | 705-605-003 | | |  |  |
| Donkey anti-mouse Cy3 |  | | 1:500 | | | Jackson | | | 715-165-151 | | |  |  |
| Donkey anti-rabbit 488 |  | | 1:500 | | | Jackson | | | 711-545-152 | | |  |  |
| Donkey anti-rabbit AF647 |  | | 1:500 | | | Jackson | | | 711-605-152 | | |  |  |
| Donkey anti-rabbit Cy3 |  | | 1:500 | | | Jackson | | | 711-165-152 | | |  |  |
|  |  | | | |  | | |  | | |  | | |
|  |  | | | |  | | |  | | |  | | |
|  |  | | | |  | | |  | | |  | | |
|  |  | | | |  | | |  | | |  | | |
|  |  | | | |  | | |  | | |  | | |
|  |  | | | |  | | |  | | |  | | |
|  |  | | | |  | | |  | | |  | | |

**Table S5.** qPCR Primer Sequences

| **Gene** | **Forward (5’ to 3’)** | **Reverse (5’ to 3’)** | |  |
| --- | --- | --- | --- | --- |
| *AQP5* | CTG GCA TCC TCT ACG GTG T | | AGA ATC AGC TCC ACC ACC AT | |
| *αSMA* | CCG ACC GAA TGC AGA AGG A | | ACA GAG TAT TTG CGC TCC GAA | |
| *FOXA2* | CGA CTG GAG CAG CTA CTA TGC | | TAC GTG TTC ATG CCG TTC AT | |
| *GAPDH* | CTC TGC TCC TCC TGT TCG AC | | TTA AAA GCA GCC CTG GTG AC | |
| *HOPX* | GCC TTT CCG AGG AGG AGA C | | TCT GTG ACG GAT CTG CAC TC | |
| *Ki67* | TGG GTC TGT TAT TGA TGA GCC | | TGA CTT CCT TCC ATT CTG AAG AC | |
| *NANOG* | ACC TCA GCT AGA AAC AGG TGA | | CTT CTG CGT CAC ACC ATT GC | |
| *NKX2.1* | CTC ATG TTC ATG CCG CTC | | GAC ACC ATG AGG AAC AGC G | |
| *OCT4* | AGT TTG TGC CAG GGT TTT TG | | ACT TCA CCT TCC CTC CAA CC | |
| *PAX8* | TGC CTC ACA ACT CCA TCA GA | | CAG GTC TAC GAT GCG CTG | |
| *PDGFRα* | GAA GGT GGT TGA AGG AAC AGC | | AGG CTC CCA GCA AGT TTA CAA | |
| *PDPN* | ACA TCC TTT GTT TTT GCC CA | | AGT GTC ATC TTC TGG CTG GC | |
| *SFTPB* | GAG CCG ATG ACC TAT GCC AAG | | AGC AGC TTC AAG GGG AGG A | |
| *SFTPC* | AGC AAA GAG GTC CTG ATG GA | | CGA TAA GAA GGC GTT TCA GG | |
| *SOX17* | TCT GCC TCC TCC ACG AAG | | CAG AAT CCA GAC CTG CAC AA | |
| *SOX2* | GCT TAG CCT CGT CGA TGA AC | | AAC CCC AAG ATG CAC AAC TC | |
| *SOX9* | GTA CCC GCA CTT GCA CAA C | | GTG GTC CTT CTT GTG CTG C | |
| *VIM* | CTT CAG AGA GAG GAA GCC GA | | ATT CCA CTT TGC GTT CAA GG | |
