# Supplementary figures and images for: Human induced pluripotent stem cell‐derived lung organoids in an ex vivo model of the congenital diaphragmatic hernia fetal lung
Source: Stem Cells Transl Med. 2020 Sep 19;10(1):98–114. doi: 10.1002/sctm.20-0199 (PMC7780804; doi:10.1002/sctm.20-0199)

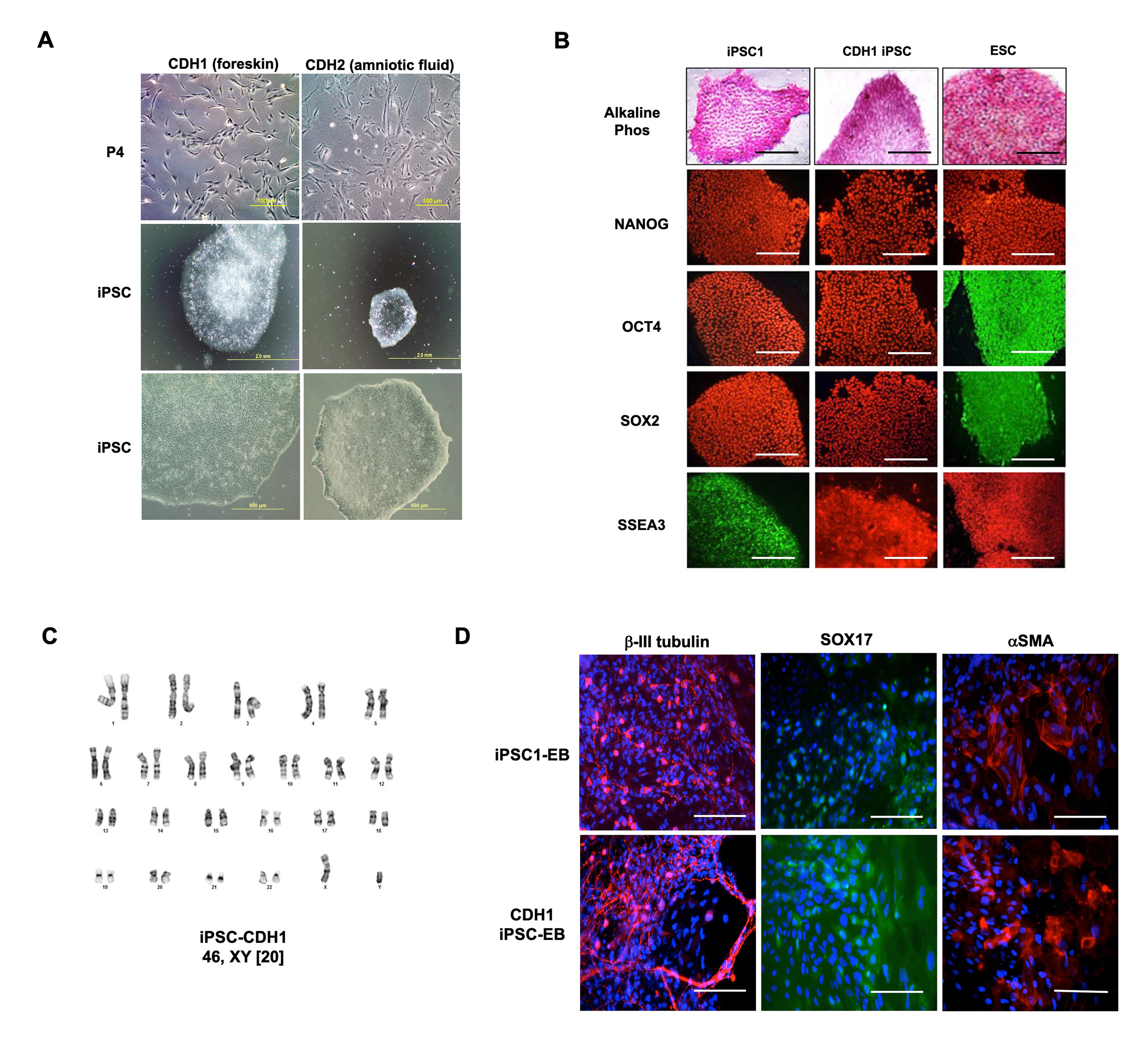

Supplement: Supplementary file 2 — Figure S1 Generation of human pluripotent stem cells derived from congenital diaphragmatic hernia (CDH) patients. (A) Representative phase contrast photomicrographs of foreskin and amniotic fluid mesenchymal cells at passage 4 (upper panels, magnification, 60x) prior to reprogramming. Representative colony morphology of induced pluripotent stem cells (iPSCs) at 10x (middle panels) and 40x (lower panels) magnification after exposure to Sendai virus. (B) Representative alkaline phosphatase staining and immunofluorescence profile of CDH iPSCs shows marked similarities with iPSCs from children with normal lungs as well as human embryonic stem cell (ESC) controls (magnification, 20x). Scale bars represent 100 μm. (C) Representative karyotype analysis of iPSC colonies (passage 10) derived from neonatal foreskin shows normal chromosomes based on twenty 20 G‐banded metaphase cells. (D) Immunofluorescence microscopy of representative adherent embryoid bodies (EB) from normal and CDH iPSCs demonstrates similar spontaneous three germ layer expression of β‐III tubulin (ectoderm), SOX17 (endoderm), and αSMA (mesoderm) superimposed with DAPI, magnification, 40x. Scale bars represent 50 μm. [file SCT3-10-98-s002.tif]

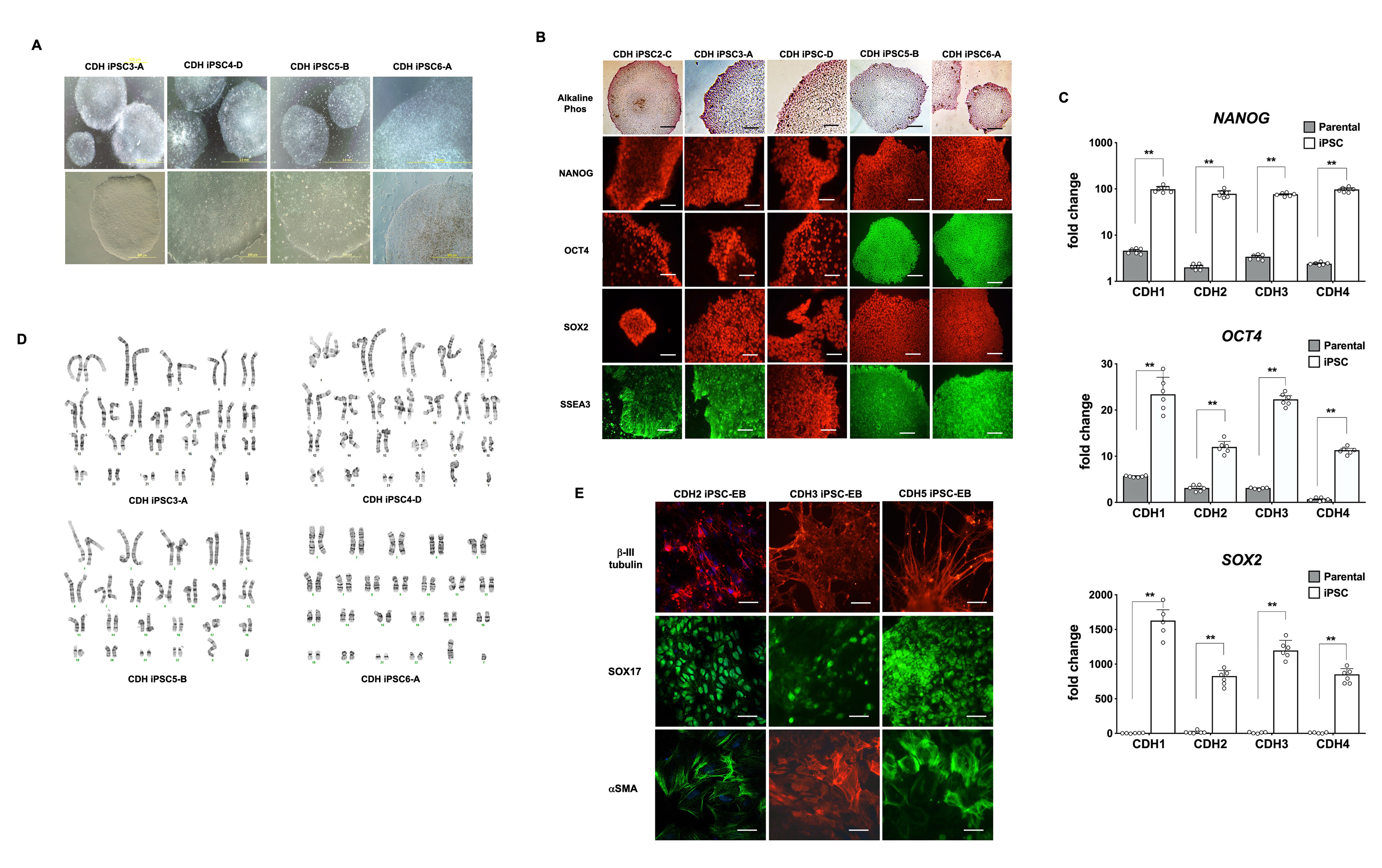

Supplement: Supplementary file 3 — Figure S2 Characterization of additional clones derived from human pluripotent stem cells from congenital diaphragmatic hernia (CDH) patients. (A) Representative phase contrast photomicrographs of colony morphology of induced pluripotent stem cells (iPSCs) at 10x (top panels) after exposure to Sendai virus. (B) Representative alkaline phosphatase staining and immunofluorescence profile of CDH iPSCs shows marked similarities with iPSCs from children with normal lungs as well as human embryonic stem cell (ESC) controls (magnification, 20x). Scale bars represent 100 μm. (C) Vertical bar graphs with dot plots demonstrate significant upregulation of pluripotency‐specific genes, including NANOG, OCT4, and SOX2, in iPSC clones (shown in white) derived from four different CDH patients (CDH1‐CDH4, passage 22‐26) compared to that in respective parental cell controls (passage 4). Data were normalized relative to housekeeping gene (GAPDH) and presented as the mean ± SEM, ** denotes P ≤ 0.01 compared to control (Mann‐Whitney). (D) Representative karyotype analysis of iPSCs colonies (passages 8‐14) derived from neonatal foreskin or amniotic fluid shows normal chromosomes based on twenty 20 G‐banded metaphase cells. (E) Immunofluorescence microscopy of representative adherent embryoid bodies (EB) from normal and CDH iPSCs demonstrates similar spontaneous three germ layer expression of β‐III tubulin (ectoderm), SOX17 (endoderm), and αSMA (mesoderm) superimposed with DAPI (magnification, 40x). Scale bars represent 50 μm. [file SCT3-10-98-s003.tif]
